# Transplantation of neuron‐inducing grafts embedding positively charged gold nanoparticles for the treatment of spinal cord injury

**DOI:** 10.1002/btm2.10326

**Published:** 2022-04-18

**Authors:** Wan‐Kyu Ko, Seong Jun Kim, Gong Ho Han, Daye Lee, Dabin Jeong, Sang Jin Lee, In‐Bo Han, Je Beom Hong, Seung Hun Sheen, Seil Sohn

**Affiliations:** ^1^ Department of Neurosurgery CHA Bundang Medical Center, CHA University Seongnam‐si Gyeonggi‐do Republic of Korea; ^2^ Department of Biomedical Science CHA Bundang Medical Center, CHA University Seongnam‐si Gyeonggi‐do Republic of Korea; ^3^ Department of Biology Lawrence University Appleton Wisconsin USA; ^4^ Department of Dental Materials, School of Dentistry Kyung Hee University Seoul Republic of Korea; ^5^ Department of Neurosurgery Kangbuk Samsung Hospital, Sungkyunkwan University School of Medicine Seoul Republic of Korea

**Keywords:** astrocyte, gold nanoparticle, neural stem cell, neuron, spinal cord injury

## Abstract

In this study, we aimed to investigate the recovery after traumatic spinal cord injury (SCI) by inducing cellular differentiation of transplanted neural stem cells (NSCs) into neurons. We dissociated NSCs from the spinal cords of Fisher 344 rat embryos. An injectable gel crosslinked with glycol chitosan and oxidized hyaluronate was used as a vehicle for NSC transplantation. The gel graft containing the NSC and positively charged gold nanoparticles (pGNP) was implanted into spinal cord lesions in Sprague–Dawley rats (NSC‐pGNP gel group). Cellular differentiation of grafted NSCs into neurons (stained with β‐tubulin III [also called Tuj1]) was significantly increased in the NSC‐pGNP gel group (***p < 0.001) compared to those of two control groups (NSC and NSC gel groups) in the SCI conditions. The NSC‐pGNP gel group showed the lowest differentiation into astrocytes (stained with glial fibrillary acidic protein). Regeneration of damaged axons (stained with biotinylated dextran amines) within the lesion was two‐fold higher in the NSC‐pGNP gel group than that in the NSC gel group. The highest locomotor scores were also found in the NSC‐pGNP gel group. These outcomes suggest that neuron‐inducing pGNP gel graft embedding embryonic spinal cord‐derived NSCs can be a useful type of stem cell therapy after SCI.

## 1 INTRODUCTION

Traumatic spinal cord injury (SCI) results in severe functional impairment accompanied by a loss of neurons at the injury site.^1^ Although multiple neurotrophic factors have been administered to lesions in an effort to improve the recovery of injured neurons, damaged neurons rarely recover due to their poor regenerative capability.^2^ Instead, several stem cells such as neural stem cells (NSC) dissociated from embryos, and induced pluripotent stem cells have been transplanted into the injured spinal cord to rectify the neuronal loss.^3^, ^4^, ^5^ Specifically, dissociated NSCs from the spinal cords of embryos are quite promising candidates in clinics because NSCs have pluripotency and can thus differentiate into neurons.^6^, ^7^, ^8^, ^9^ Indeed, human embryonic spinal cord‐derived NSCs, including NSI‐566 cells have been tested in SCI patients for safety.^6^

Gold nanoparticles (GNP) are quite attractive biomedical materials owing to their unique characteristics, including nonimmunogenic and nontoxic biocompatibility.^10^, ^11^, ^12^ In addition, the GNPs, including gold nanorods have been successfully employed to increase the neurite outgrowth of neurons under laser irradiation.^13^, ^14^ In the present study, we investigate whether GNP can induce the neuronal differentiation of embryonic spinal cord‐derived NSCs. To increase the amount of endocytosed GNP into the NSC, positively charged gold nanoparticles (pGNP) were embedded in the NSC graft. The positive charge is favorable for attachment onto cell membranes, as electrical force can be generated between the pGNP and negatively charged cell membranes.^15^

Chitosan was approved by the Food and Drug Administration (FDA) for its usage in tissue engineering.^16^ Hyaluronate also was approved by the FDA as a bioinert material.^17^ In this study, we used a hydrogel composed of glycol chitosan (gC) and oxidized hyaluronate (oHA) as a vehicle for the transplantation of NSCs. In our recent works, we demonstrated that gC‐oHA (CHA) gel has fully degradable property.^18^, ^19^ In this study, allogeneic animal models were used. CHA gel grafts containing the pGNP, and green fluorescent protein (GFP)‐expressing NSCs were prepared. The NSCs were dissociated from embryonic day 14 (E14) spinal cords of Fisher 344 (F344) rats. The graft was transplanted into spinal cords severely injured through a contusion method involving Sprague–Dawley (SD) rats (NSC‐pGNP gel group). In this study, we transplanted the NSC grafts at 2 weeks after SCI for injection at a sub‐acute phase (7–21 days after injury).^20^ The NSCs rarely survived during the acute phase (0–7 days after injury) due to the production of inflammatory cytokines following the injury.^21^

## 2 MATERIALS AND METHODS

### 2.1 Materials and reagents

Chloroauric acid (HAuCl_4_), branch polyethyleneimine (bPEI), trisodium citrate, gC (molecular weight [MW]: 50,000 Da), and sodium periodate (NaIO_4_) were purchased from Sigma Aldrich. Sodium hyaluronate (HA, MW: 1000 kDa) was provided by Humedix. Deionized water (DW, 18.2 MΩ) was prepared using an EXL‐3 water purification system (Vivagen). Neurobasal™ plus medium (GIBCO, Life Technologies) containing the B27 supplement and 1% penicillin–streptomycin (PS, GIBCO) (cell culture medium [CCM]) was used for culturing the E14‐derived NSCs. Dulbecco's phosphate‐buffered saline (DPBS) was also purchased from GIBCO. The 100‐mm and 48‐well cell culture plates used here were both purchased from Falcon Becton Dickinson (Falcon).

### 2.2 Preparation of negatively charged GNPs (nGNP) and pGNP

The nGNP was prepared as previously described.^22^, ^23^ Detailed descriptions of the synthesis methods are provided in the Supporting Information S1. The pGNP was prepared as described.^24^ Briefly, HAuCl_4_ powder (9.72 mg) was dissolved in 200 μl of DW (9.72 mg/200 μl: solution 1). Next, 350 mg of bPEI was diluted in 5 ml of DW to obtain 70 mg/ml of a PEI solution, after which 87.45 μl of the 70 mg/ml PEI was transferred to 10 ml of fresh DW (solution 2). Solution 1 was slowly dropped into solution 2 and gently stirred with a magnetic bar for 7 d at room temperature. Afterwards, the dark red solution was stored at 4°C before use. The concentration of the synthesized pGNP was 2.49 nM, as estimated by the Beer–Lambert law with a molar extinction coefficient of 3.36 × 10^9^ M^−1^ cm^−1^.^25^

### 2.3 CHA gel preparation

The cell transplantation vehicle, CHA gel, was prepared as previously described.^18^, ^19^ Two % weight per volume (w/v) of gC (2% gC) and 3% w/v of oHA (3% oHA) were dissolved in separate amounts of DPBS. The volume ratio of the 2% gC and 3% oHA to form the CHA gel was 9:1. The gC and oHA were stored at −20°C before use. Detailed preparation methods for the 2% gC and 3% oHA are given in the Supporting Information S1.

### 2.4 Characterization of the nGNP and pGNP

GNP concentrations were determined from Ultraviolet/visible (UV/vis) absorption values as measured by a spectrophotometer (UV‐1650PC, Shimadzu). The absorbance levels of both nGNP and pGNP (at 0.05, 0.1, 0.2, and 0.3 nM) were measured by a UV/vis spectrophotometer as well. The size distributions of the synthesized nGNP and pGNP were evaluated by dynamic light scattering (DLS, Malvern 4700). The zeta potential (Zetasizer 2000) levels were measured to investigate the surface charges of the nGNP and pGNP at a concentration of 0.1 nM.

### 2.5 Animals

A total of 54 adult female rats, including SD (*n* = 12, KOATECH) and F344 rats (*n* = 42, Rat Resource and Research Center, University of Missouri) were used in this study. Twelve female SD rats (210–240 g) underwent spinal cord contusion. Forty‐two pregnant F344 rats, ubiquitously expressing GFP, were sacrificed to harvest embryonic tissues. The rats were housed in a facility at 55%–65% humidity and a controlled temperature of 24°C ± 3 with a light/dark cycle of 12 h and were given free access to water/food. All surgical interventions and postoperative animal care procedures were performed in accordance with the Guidelines and Polices for Rodent Survival Surgery provided by the Institutional Animal Care and Use Committee (IACUC) of CHA University (IACUC 200119). National Institutes of Health (NIH) guidelines for laboratory animal care and safety were also followed.

### 2.6 NSC dissociation for a cytotoxicity test

The E14 spinal cords (expressing GFP) were dissociated as described in the literature.^8^, ^9^ The spinal cord dissociation process is described as follows: spinal cords were digested in 0.25% trypsin (GIBCO) for 10 min at 37°C, dissociated in CCM, filtered using a 40 μm cell filter strainer, centrifuged, and then resuspended. The cells were seeded on 48‐well cell culture plates at a density level of 2 × 10^5^ and cultured with CCM for 2 days. The cytotoxicity assay was performed using a cell viability assay kit (EZ‐Cytox, Daeil Labservice). The cells were cultured with CCM containing 0, 0.05, 0.1, 0.2, or 0.3 nM of nGNP or pGNP. After 48 h, the CCM was replaced with a fresh medium containing a cell viability kit solution (500 μl of 0.1 ml/mL, *n* = 6). After incubation for 1 h, the absorbance was measured at 450 nm with a microplate reader (Bio‐Rad). The absorbance of the without GNP group at 48 h was fixed at 100% to normalize the absorbance levels of the other groups.

### 2.7 Immunofluorescence staining and quantification in vitro

The dissociated NSCs from the spinal cords were seeded on 20‐mm confocal dishes (SPL, Korea, *n* = 9) at a density level of 4 × 10^5^ and cultured with CCM for 1 day. The nine confocal dishes used were randomly divided into three experimental groups (*n* = 3 per group). After 1 and 4 days, the medium was replaced with a fresh medium without GNP (without GNP group), with 0.1 nM of nGNP (nGNP group), or with 0.1 nM of pGNP (pGNP group). At 6 days from the initial cell seeding, the NSCs were fixed with 4% paraformaldehyde for 20 min. They were then washed with DPBS three times and immersed in 0.2% triton X‐100 for 10 min. After washing three times with DPBS, the cells were blocked with a blocking solution (Invitrogen). Subsequently, immunofluorescence (IF) staining was performed. To label the dissociated NSC, the cells were stained with anti‐GFP (mouse, Invitrogen at 1:200). Each GFP‐stained cell was co‐stained with the β‐tubulin III (also called Tuj1, a neuron marker, rabbit, Invitrogen at 1:200), glial fibrillary acidic protein (GFAP, an astrocyte marker, rabbit, Invitrogen at 1:200), or oligodendrocyte specific protein (OSP, an oligodendrocyte marker, rabbit, Invitrogen at 1:200) antibody to evaluate the cellular differentiation into neurons, astrocytes, or oligodendrocytes, respectively. The cells were incubated overnight at 4°C and then incubated in Alexa 488‐conjugated goat or 568‐conjugated donkey secondary antibodies (both 1:500, Invitrogen) for 2 h at RT.

For each confocal dish, the regions of interest (ROIs, 425 × 425 μm^2^) were randomly designated (*n* = 9 per group) at 20× magnification. Within ROI image (scale bar: 50 μm), GFP stained area was normalized to 100%. Tuj1, GFAP, or OSP‐stained area relative to GFP‐stained area within the ROI image was quantified using the ImageJ software (NIH). The cells were detected using a confocal laser‐scanning microscope (LSM 880, Carl Zeiss).

### 2.8 Quantitative real‐time polymerase chain reaction

The quantitative real‐time polymerase chain reaction (qRT‐PCR) were performed as described previously.^26^, ^27^ The expression of Tuj1, oligodendrocyte transcription factor 2 (Olig2), and GFAP were assessed by the qRT‐PCR. The relative expression values of target genes were normalized to glyceraldehyde 3‐phosphate dehydrogenase (GAPDH) using $2^{-\Delta \Delta C_{T}}$ method.^28^ Detailed descriptions of the qRT‐PCR are provided in the Supporting Information S1.

### 2.9 The measurements and quantification of GNP uptake into NSCs

The dissociated NSCs from the spinal cords were also seeded onto 20‐mm confocal dishes (SPL, *n* = 8) at a density level of 2 × 10^5^ and cultured with CCM for 1 day. In total, eight confocal dishes were randomly divided into two experimental groups (0.1 nM of nGNP and 0.1 nM of pGNP groups, four dishes per group). At 8 and 24 h, two dishes were fixed with 4% paraformaldehyde. The particles inside the NSCs were visualized using a 12‐bit charge coupled device camera equipped with a special C‐mount lens (Digital Imaging Systems) at 100× magnification (scale bar: 20 μm). The ROI (180 × 100 μm^2^) was randomly designated (*n* = 2 per dish). Within the ROIs, the whole cell area was designated as 100%. The GNP‐positive area relative to the whole cell area was quantified using ImageJ software (NIH).

### 2.10 Contusion SCI in SD rats

SD rats (*n* = 12) underwent T9 spinal cord contusion injures (RWD, spinal cord impactor, Cat: #68097), as previously described.^18^, ^29^ Briefly, SD rats were anesthetized via an intraperitoneal injection of a combination of 10 mg/kg of Rompun (Bayer Animal Health Co) and 50 mg/kg of Zoletil 50 (Virbac Laboratories). A midline incision was made in the back. Tissues were dissected layer by layer to reveal the T8‐T10 vertebrae. A T9 total laminectomy was performed to expose the dura. The spinous processes were fixed by clamps. The exposed dorsal surface of the cord was subjected to weight‐drop impact using a 40‐gram rod (2.5 mm in diameter) from a height of 60.0 mm. The surgical site was closed layer by layer. The rats were kept warm and housed separately with free access to food. The bladders of the injured rats were manually emptied several times daily until normal function returned.

### 2.11 Transplantation surgeries of NSC grafts into SD rats

After 2 weeks from the contusion SCI, a clinically relevant time point, we dissociated E14 spinal cords from F344 rats. Dissociated E14 cells were resuspended at a concentration of 4 × 10^5^ cells/μl in a CHA gel. The pGNP concentration of 2.49 nM was centrifuged at 14,000 RPM for 10 min to prepare for the 0.1 nM of pGNP pellet. The pGNP pellet was mixed with the NSC gel graft and injected into the SD rats of the NSC‐pGNP gel group.

Twelve of the injured rats were randomly divided into three experimental groups with four animals in each. NSC suspensions with saline (NSC group), gels (NSC gel group), and pGNP‐gel (NSC‐pGNP gel group) were transplanted into the target lesion. The NSC mixture was injected to four target sites through a 31‐gauge beveled needle attached to a Hamilton syringe (Hamilton). Five microliters of NSC grafts were injected into each site. GFAP‐stained margins in host specimens including grafted GFP‐stained areas were determined as an injury area. Four weeks after the post‐NSC grafting step, reticulospinal tract axons were labeled by an injection of 0.5 μl of 10% biotinylated dextran amine (BDA, MW: 10,000, Molecular Probes) into each of two sites (±0.6 mm lateral to midline; depth: 1.5 mm) using a 33‐gauge Hamilton syringe), and the subjects survived for another 2 weeks.

### 2.12 Tissue preparation for IF staining and quantification

Two weeks after the BDA injection, 12 SD rats (four rats per group) were perfused for the evaluation of IF staining as previously described.^30^, ^31^ Serial longitudinal sections through the dorsoventral axis of the spinal cord (5 μm thick) were collected at lengths of 180 μm (36th), 185 μm (37th), 190 μm (38th), 195 μm (39th), 200 μm (40th), 205 μm (41st), 210 μm (42nd), and 215 μm (43rd) per rat. The sections were stained with anti‐GFP (rabbit, Invitrogen at 1:200). Each GFP‐stained section was co‐stained with GFAP (mouse, Invitrogen at 1:200 for the 36th and 40th), Tuj1 (mouse, Invitrogen at 1:200 for the 37th and 41st), or OSP (mouse, Invitrogen at 1:200 for the 38th and 42nd) antibody to evaluate the cellular differentiation of the grafted NSCs. To label reticulospinal tract axons, the 39th and 43rd sections were stained with BDA. The 39th and 43rd sections were incubated with Alexa 594‐conjugated streptavidin (to bind to BDA‐labeled reticulospinal tract axons) for 2 h at RT. The other sections were incubated overnight at 4°C and then incubated in Alexa 488‐conjugated goat and 568‐conjugated donkey secondary antibodies (both 1:500, Invitrogen) for 2 h at RT.

For each section, the lesion including four NSC transplanted sites was designated as the ROI (composite tiled scan images, 4500 × 2000 μm^2^, scale bar: 500 μm, *n* = 8 per group). The ROI images were detected using a confocal laser‐scanning microscope (LSM 880, Carl Zeiss). Within the injury area of the ROI images, the GFP‐stained area was designated as 100%. GFAP, Tuj1, and OSP‐stained areas relative to the GFP‐stained area were quantified using ImageJ software (NIH). BDA‐stained areas relative to the IAs were also quantified using the ImageJ software (NIH). The detailed description for the IF staining/quantification using GFAP/CD68, GFP/Tuj1/Synapsin (Syn), GFP/Tuj1/myelin basic protein (MBP), GFP/Tuj1/neuronal nuclei (NeuN), Tuj1/OSP/GFAP/GFP, GFP/neurofilament (NF)/PGP9.5, GFP/Tuj1/growth associated protein (GAP) 43, and GFP/CD68/ionized calcium‐binding adaptor molecule 1 (iba1) markers is demonstrated in the Supporting Information S1.

### 2.13 Behavioral tests

To investigate the recovery of the hindlimb locomotor function in injured SD rats, Basso, Beattie, and Bresnahan (BBB) scores were measured in an open‐field area for 56 days after the contusion injury. The BBB is a 22‐point scale (with scores of 0–21) that systematically and logically follows the recovery of the hindlimb function from a score of 0, indicative of no observed hindlimb movements, to a score of 21, representative of a normal ambulating rodent.^32^ Two trained investigators evaluated the behavioral scores in a blinded manner. All the SD rats (*n* = 4 per group) were observed at 1, 14, 21, 28, 35, 42, 49, and 56‐day post injury (DPI). The comparisons among the NSC, NSC gel, and NSC‐pGNP gel groups at the pre‐determined time point were conducted with a one‐way analysis of variance (ANOVA), and Tukey's multiple‐comparison test was used as a post‐hoc analysis method. Differences with *p*‐values for which *p < 0.05, **p < 0.01, ***p < 0.001, ^#^ *p* < 0.05, ^##^ *p* < 0.01, ^$$^ *p* < 0.01, and ^$$$^ *p* < 0.001 were considered statistically significant.

### 2.14 Statistical analysis

Multiple comparisons among the three groups were performed with a one‐way ANOVA, and Tukey's multiple‐comparison test was used as a post‐hoc analysis method. Two‐group comparisons were conducted with Student's t‐tests. Differences with *p*‐values for which *p < 0.05, **p < 0.01, and ***p < 0.001 were considered statistically significant.

## 3 RESULTS

### 3.1 Characteristics of synthesized nGNP and pGNP

**FIGURE 1:**
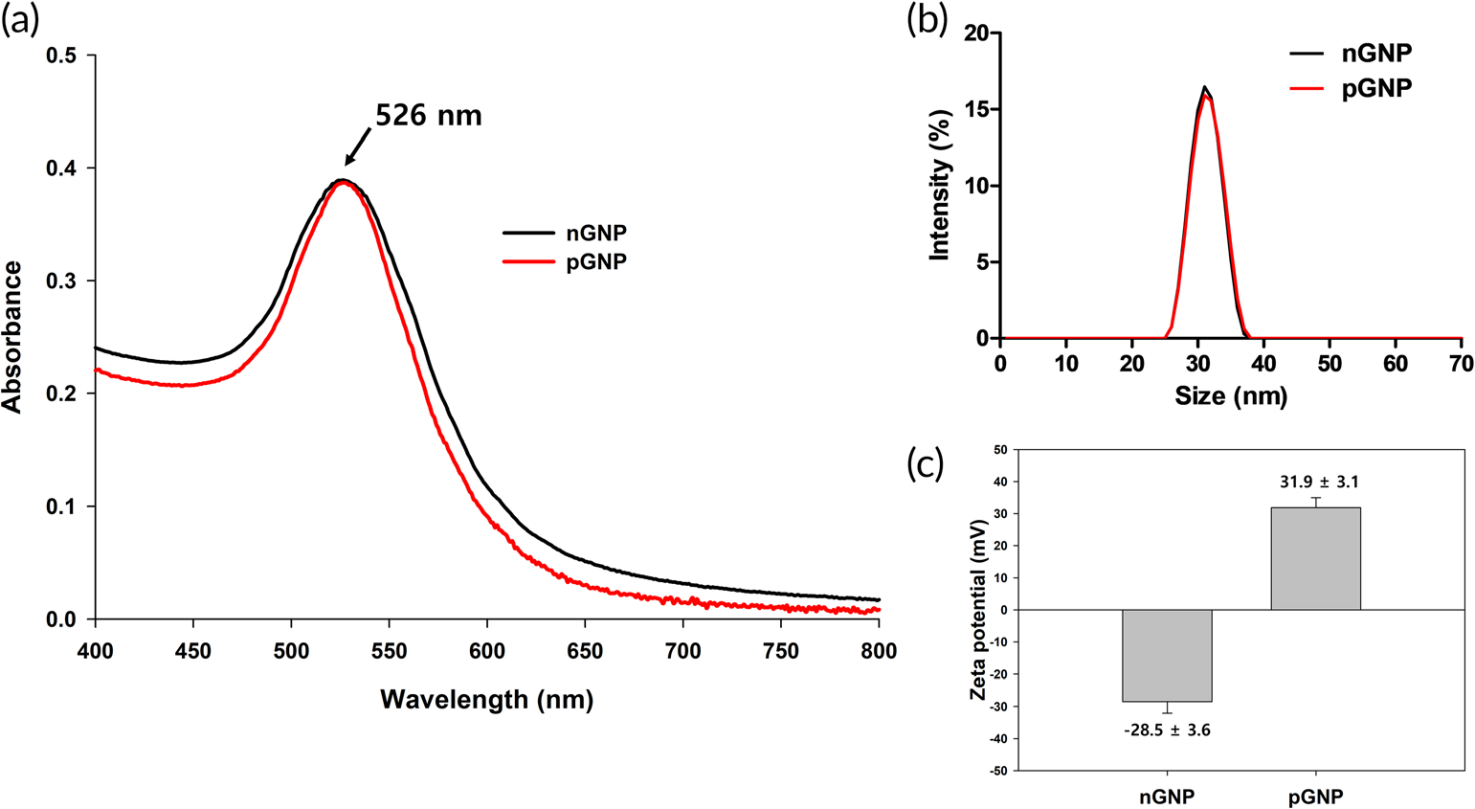
Characterization of negatively charged gold nanoparticles (nGNP) and positively charged gold nanoparticles (pGNP) at 0.1 nM. (a) Absorbance values ranged from 400 to 800 nanometer (nm) for both GNPs. (b) Dynamic light scattering analysis of both GNPs for the measurement of the size distribution. (c) Surface charge measurements for both GNPs using the zeta potential

Both nGNP and pGNP showed a peak at 526 nm in the wavelength range of 400–800 nm (Figure 1a). The peak at 526 nm demonstrated that both the nGNPs and pGNPs are approximately 30 nm in size.^25^ GNPs that are 30 nm in size are optimal for attachment onto cell membranes and endocytosis into cells.^19^ The two types of GNPs had a diameter distribution in the range of 25–36 nm (Figure 1b). The surface charge of the synthesized nGNP was −28.5 mV ± 3.6, while the charge of the pGNP was 31.9 mV ± 3.1 (Figure 1c).

### 3.2 Cytotoxicity tests of nGNP and pGNP

The cell viability rates of the nGNP‐treated group were 100% ± 2.8, 99.3% ± 2.7, 97.7% ± 5.1, 93.2% ± 3.8, and 84.2% ± 3.3 at 0, 0.05, 0.1, 0.2, and 0.3 nM, respectively (Figure S1a). For the pGNP group, the rates were 100% ± 3.0, 100.9% ± 3.1, 103.6% ± 2.3, 94.1% ± 3.0, and 83.3% ± 3.6 at 0, 0.05, 0.1, 0.2, and 0.3 nM, respectively (Figure S1b). The cell viability of both GNP groups rarely decreased up to a concentration of 0.1 nM. However, the rates started to slightly decrease from 0.2 nM. Therefore, a 0.1 nM concentration of GNP was used to investigate whether GNP with a negative or positive charge would affect NSC differentiation.

### 3.3 The differentiation rate into neurons was increased in the order of the without (w/o) GNP, nGNP, and pGNP groups in vitro

**FIGURE 2:**
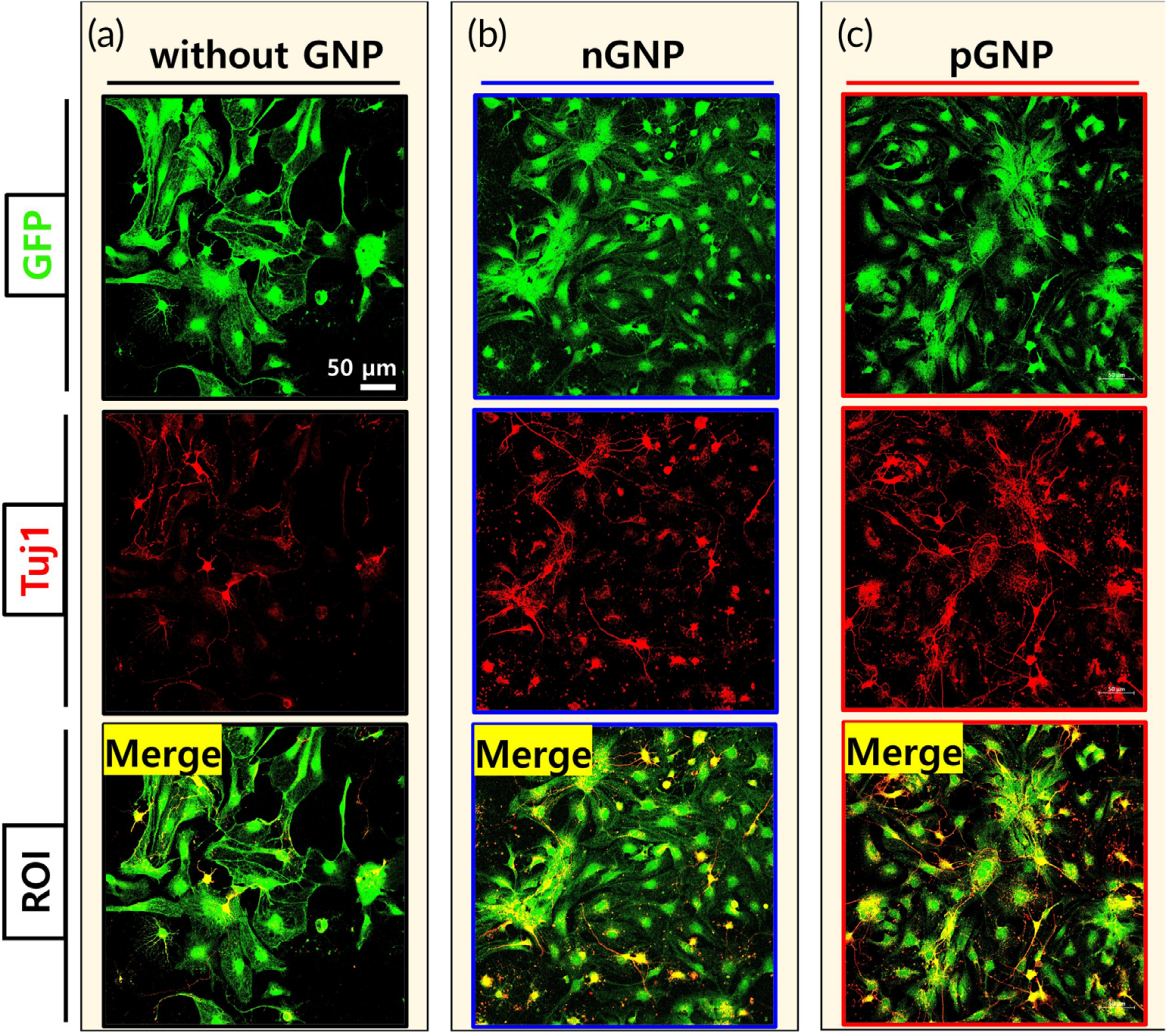
Cellular differentiation of green fluorescent protein (GFP)‐expressing neural stem cells. The cells were cultured for 6 days without charged gold nanoparticles (GNP), with negatively charged gold nanoparticles (nGNP) (0.1 nM), or with positively charged gold nanoparticles (pGNP) (0.1 nM). On day 6, the cells were co‐stained with GFP and β‐tubulin III (Tuj1). Representative images (also designated as the regions of interest, 425 × 425 μm^2^) of the groups (a) without GNP, (b) with nGNP, and (c) with pGNP were demonstrated at 20× magnification. Scale bar is 50 μm

**FIGURE 3:**
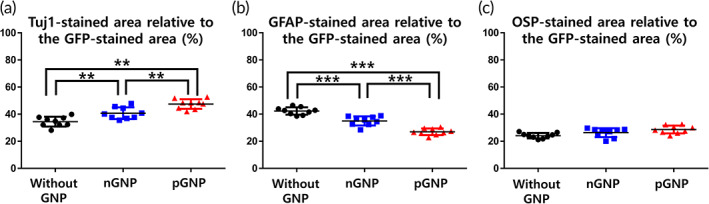
Cellular differentiation of the dissociated neural stem cells in the regions of interest was quantified (*n* = 9 per group). The green fluorescent protein (GFP)‐stained area in each case was normalized to 100%. (a) Tuj1, (b) glial fibrillary acidic protein, or (c) oligodendrocyte specific protein‐stained area relative to the GFP‐stained area was quantified using ImageJ software. Results are the mean ± standard deviation (SD): **p < 0.01 and ***p < 0.001; significant differences among the three groups were demonstrated. Multiple comparisons among the three groups were performed with a one‐way analysis of variance (ANOVA), and Tukey's multiple‐comparison test was used as a post‐hoc analysis method

Co‐stained areas in the ROI images were considered as differentiated cells from the NSCs (Figures 2, S2, and S3). As shown in the ROI images, the pGNP group exhibited the highest expression of Tuj1 (Figure 2c). The Tuj1 rates were increased in the order of the w/o GNP, nGNP, and pGNP groups (34.5% ± 3.6, 40.7% ± 4.3, and 47.5% ± 3.6, respectively, **p < 0.01, Figure 3a). Figure S2 presents ROI images of GFAP (an astrocyte marker) stained area. The GFAP rates were 42.3% ± 2.7, 35.0% ± 3.4, and 27.0% ± 2.4 for the w/o GNP, nGNP, and pGNP groups, respectively (***p < 0.001, Figure 3b). ROI images of OSP (an oligodendrocyte marker) stained area are provided in Figure S3. The OSP rates were 24.1% ± 2.0, 26.3% ± 3.3, and 28.6% ± 2.9 for the w/o, nGNP, and pGNP groups, respectively (Figure 3c). The highest Tuj1 rate and the lowest GFAP rate were shown in the pGNP group (Figure 3a,b).

The mRNA value of Tuj1 was highest in the pGNP gel group (Figure S4a). The Tuj1 values were 1.00 ± 0.00, 4.25 ± 0.33, and 8.28 ± 0.80 for the gel, nGNP gel, and pGNP gel groups, respectively (**p < 0.01). In addition, the value of GFAP mRNA was lowest in the pGNP gel group (Figure S4b). The GFAP values were 1.00 ± 0.00, 0.73 ± 0.11, and 0.35 ± 0.06 for the gel, nGNP gel, and pGNP gel groups, respectively (**p < 0.01).

### 3.4 The uptake amount of GNP is higher in the pGNP group than the nGNP group in vitro

**FIGURE 4:**
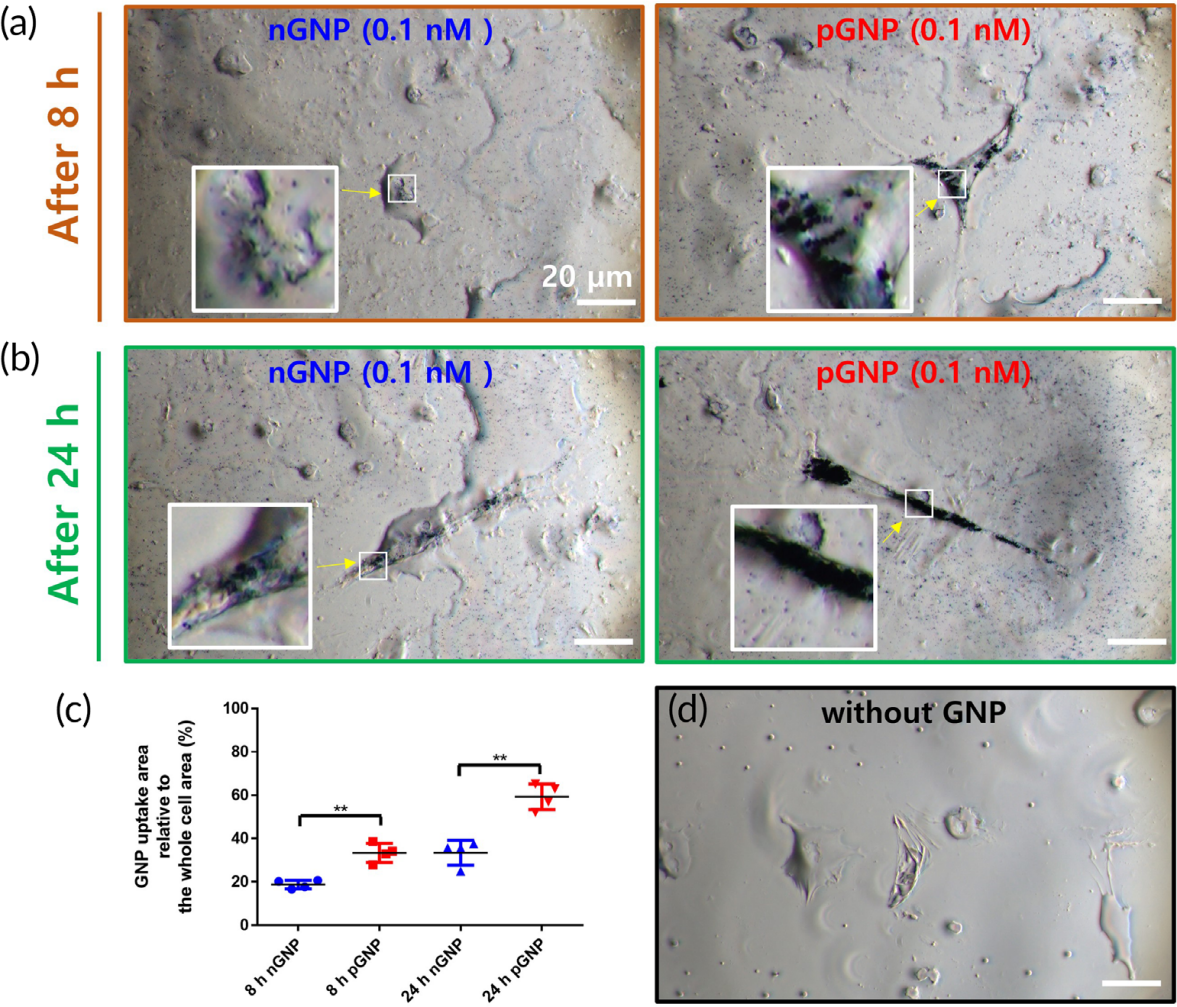
Quantification of the uptake amounts of negatively charged gold nanoparticles (nGNP) (0.1 nM) and positively charged gold nanoparticles (pGNP) (0.1 nM) into neural stem cells for 8 and 24 h. Representative images (also designated as regions of interest [ROIs], 180 × 100 μm^2^) at (a) 8 h and (b) 24 h were taken at 100× magnification. Scale bars are 20 μm. (c) Within the ROI, the whole cell area was designated as 100%. The GNP uptake relative to the whole cell area was quantified using ImageJ software. Results are the mean ± SD: **p < 0.01; significant differences between the nGNP group and the pGNP groups were analyzed by Student's *t*‐tests (*n* = 4 per group). (d) A representative image without GNPs is also shown. Arrows indicate randomly designated regions for higher magnification views

Representative images taken at 8 and 24 h are shown in Figure 4a,b, respectively. The GNP uptake rates were higher in the pGNP group (33.5% ± 5.7 and 59.3% ± 5.9 at 8 and 24 h, respectively) compared to those in the nGNP group (18.8% ± 2.0 and 33.4% ± 4.4 at 8 and 24 h, respectively) (Figure 4c). Given the higher Tuj1 marker expression level in the pGNP group (Figure 2b,c), the increased uptake amounts of pGNP imply this relative increase in Tuj1 expression (Figure 3a). Furthermore, the pGNP‐treated NSCs showed the lowest rate of astrocytic differentiation (Figure 3b). Therefore, we adopted pGNP to induce cellular differentiation into neurons from embryo derived NSCs in a contusive SCI rat model.

### 3.5 Grafted NSCs in the graft‐embedding pGNP were mainly differentiated into neurons despite the SCI condition in vivo

**FIGURE 5:**
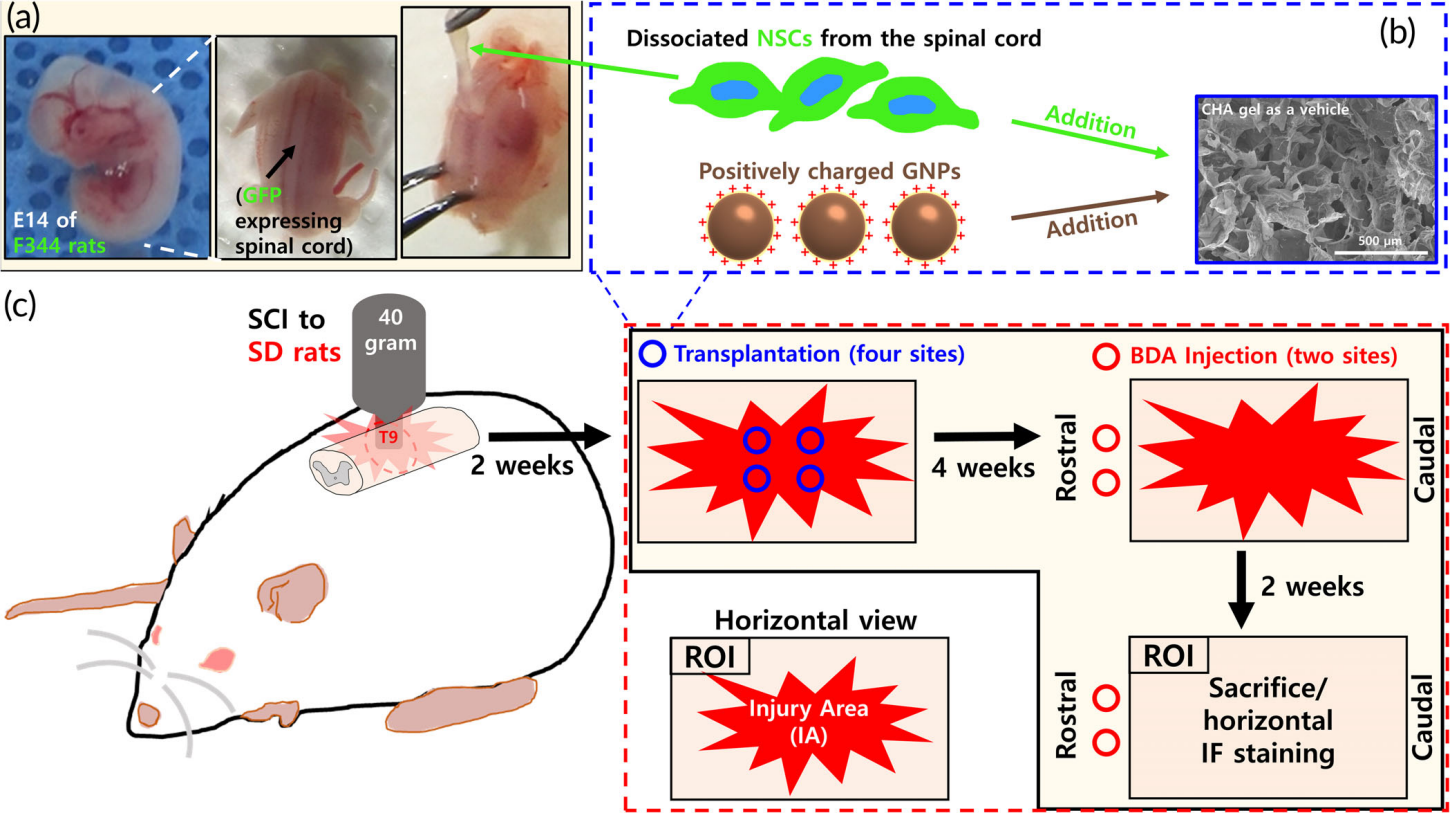
Schematic design for neural stem cell (NSC) dissociation and immunofluorescence staining. (a) Preparation of the NSC suspension from the green fluorescent protein‐expressing spinal cord. (b) NSC‐positively charged gold nanoparticles gel grafts for transplantation. (c) Contusion injury and graft transplantation process in Sprague–Dawley rats

**FIGURE 6:**
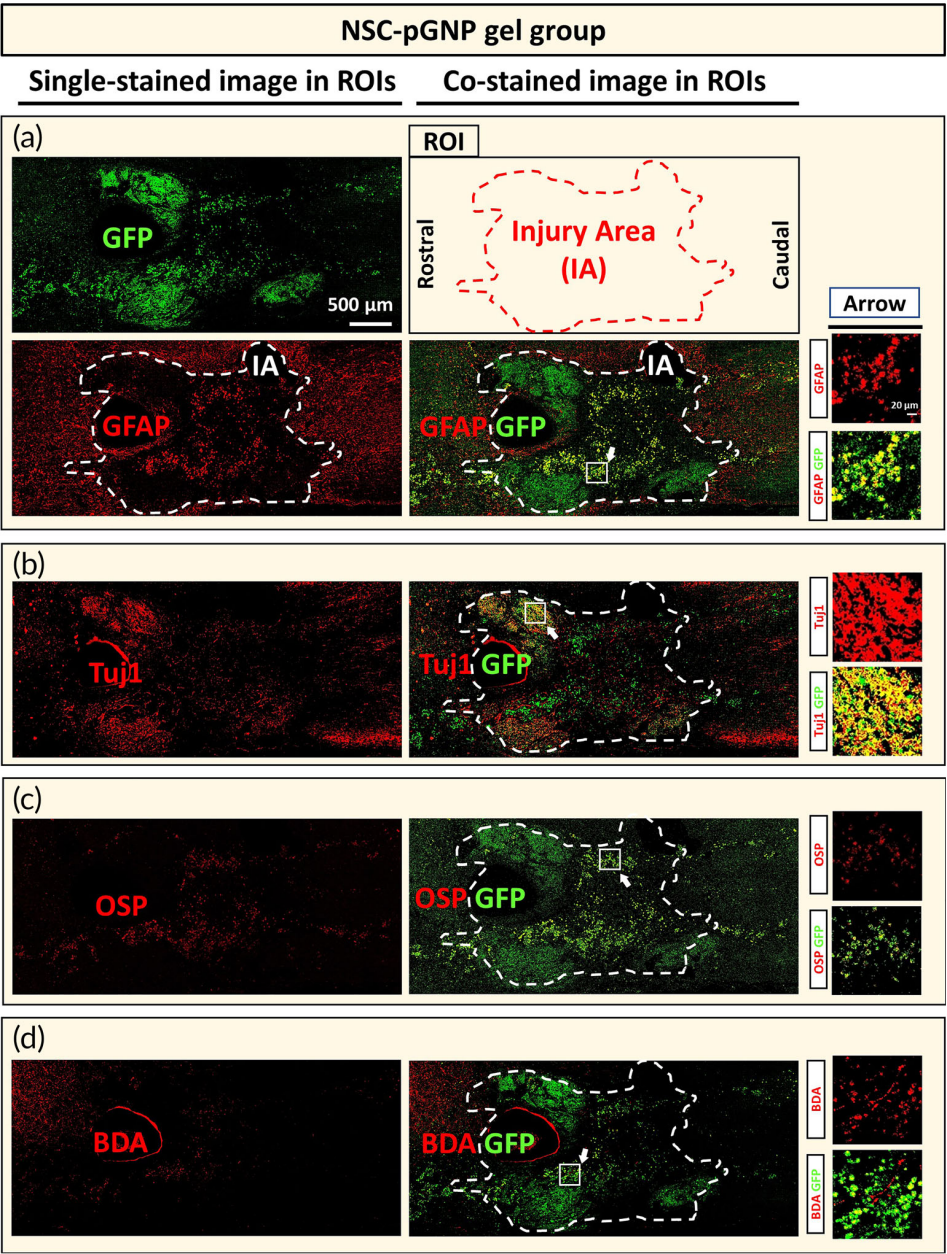
Cellular differentiation of grafted neural stem cells (NSCs) under the spinal cord injury condition for the NSC‐positively charged gold nanoparticles gel group. Horizontally sectioned specimens were stained with the green fluorescent protein (GFP) antibody. Each GFP‐stained section was co‐stained with glial fibrillary acidic protein (GFAP) (36th and 40th), Tuj1 (37th and 41st), oligodendrocyte specific protein (OSP) (38th and 42nd), or biotinylated dextran amines (BDA, 39th and 43rd). Representative tile scan images (also designated as regions of interest [ROIs], 4500 × 2000 μm^2^) of samples co‐labeled with (a) GFAP/GFP, (b) Tuj1/GFP, (c) OSP/GFP, and (d) BDA/GFP are shown. Scale bar of the ROI image is 500 μm (IA = Injury Area). Arrows indicate randomly designated regions for higher magnification views. The designated region is 170 × 170 μm^2^ (Scale bar: 20 μm)

A schematic design of the in vivo experiments is provided in Figure 5. The injury area surrounded with astrocyte barriers (stained with GFAP) is indicated by the white dashed lines (Figure 6a). We found alive NSCs in the NSC‐pGNP gel, NSC, and NSC gel groups despite SCI conditions (Figures 6, S5, S6, respectively).

**FIGURE 7:**
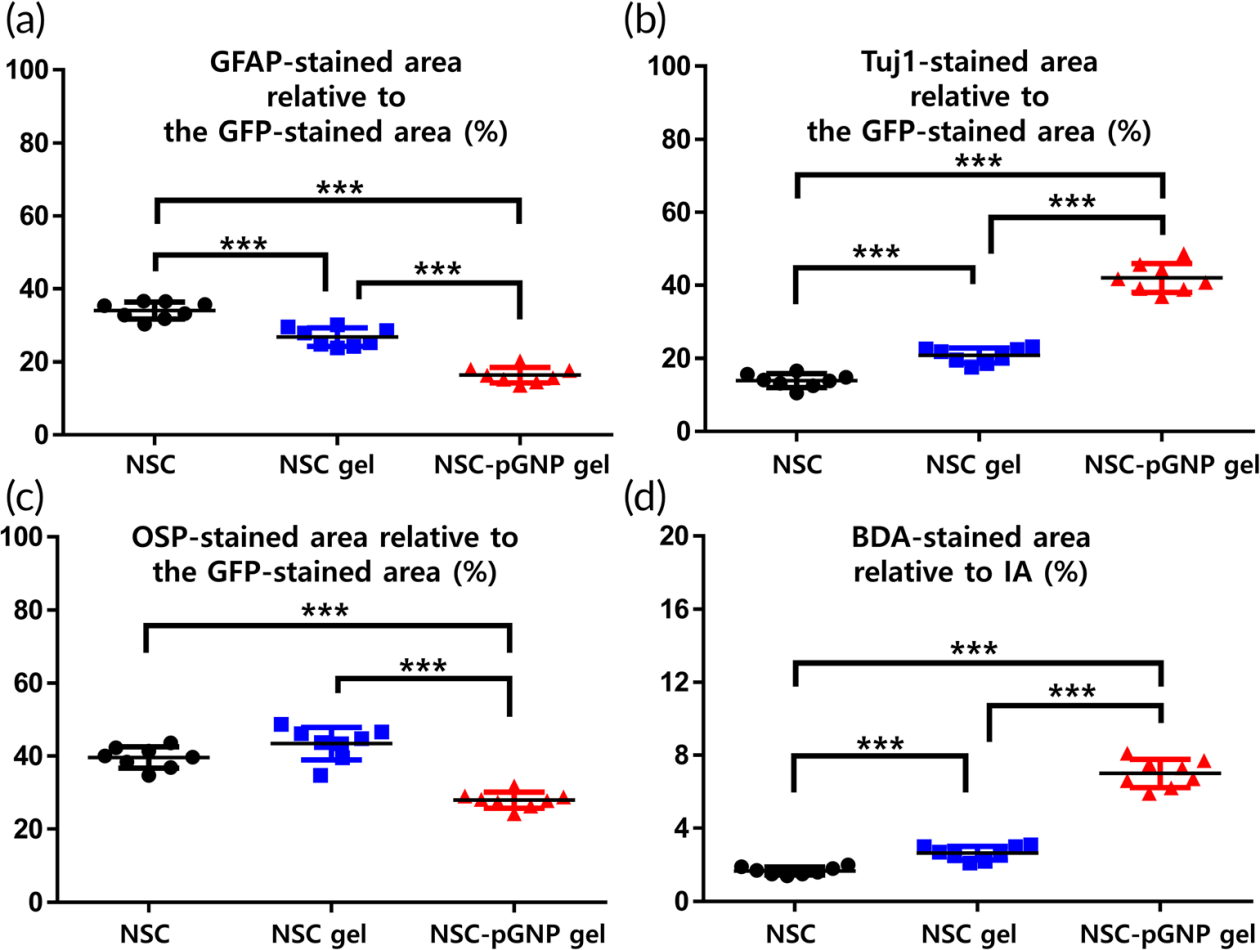
Cellular differentiation of grafted neural stem cells within IAs was quantified (*n* = 8 per group). Within the IA, the green fluorescent protein (GFP)‐stained area was normalized to 100%. (a) Glial fibrillary acidic protein, (b) Tuj1, and (c) oligodendrocyte specific protein‐stained area relative to the GFP‐stained area was quantified using ImageJ software. (d) The injury area was normalized to 100% and biotinylated dextran amines‐stained area relative to the injury area was quantified using ImageJ software. Results are the mean ± SD: ***p < 0.001; significant differences among the three groups were demonstrated. Multiple comparisons among the three groups were performed with a one‐way ANOVA, and Tukey's multiple‐comparison test was used as a post‐hoc analysis method

GFP‐stained areas within IAs of ROI images are designated as 100%. Co‐stained areas within the injury area (Figures 6, S5, S6) were quantified (GFAP, Tuj1, and OSP in Figure 7a–c, respectively). The GFAP and Tuj1 rates were 16.4% ± 2.1 and 42.0% ± 3.9 in the NSC‐pGNP gel group, respectively. The OSP rate was 27.9% ± 2.2. In the NSC group, the GFAP and Tuj1 rates were 34.0% ± 2.3 and 13.9% ± 1.9, respectively. The OSP rate was 39.6% ± 2.9. In the NSC gel group, the GFAP and Tuj1 rates were 26.8% ± 2.5 and 20.8% ± 2.0, respectively. Taken together, both the highest rate into neurons and the lowest rate into astrocytes from grafted cells were observed in the NSC‐pGNP gel group. The NSC‐pGNP gel group demonstrated a two‐fold increase in the average rate of Tuj1 expression (42.0% ± 3.9) compared to the NSC gel group (20.8% ± 2.0).

BDA (a marker of axons)‐stained areas within IAs (Figure 6d) were quantified (Figure 7d). The injury area is designated as 100%. The highest BDA rate was also shown in the NSC‐pGNP gel group. The BDA rate was 1.7% ± 0.2 in the NSC group. The average rate was increased by more than two times in the NSC‐pGNP gel group (7.0% ± 0.8, ***p < 0.001) compared to the NSC gel group (2.7% ± 0.4). The significant increases in the expression levels of Tuj1 and the BDA‐stained areas suggest that pGNP‐induced neurons promote the regeneration of injured axons. However, penetrating axons across the entire lesion border were not observed in the NSC‐pGNP gel group (Figure 6d).

### 3.6 The steadily increased BBB locomotor scores after SCI formed a plateau from 42 DPI

The average scores in three groups were steadily increased until 42 DPI. At 42 DPI, the BBB scores were 13.9 ± 0.5 and 12.5 ± 0.8 in the NSC‐pGNP gel group and in the NSC gel group, respectively. After 42 DPI, the scores in the three groups were maintained until the sacrifice day. The average score was slightly increased in the NSC‐pGNP gel group (14.0 ± 0.8) compared to that in the NSC gel group (12.8 ± 0.3) at 56 DPI. The score was lowest in the NSC group at 56 DPI (10.6 ± 0.5).

### 3.7 The decreased injury area due to the transplantation of NSC‐pGNP gel graft

The rates of injury areas were 61.6% ± 3.7, 51.2% ± 3.9, and 50.4% ± 5.2 in the NSC, NSC gel, and NSC‐pGNP gel groups, respectively (Figure S7a–d). The rates of CD68‐stained areas were 15.1% ± 2.5, 5.7% ± 1.2, and 6.2% ± 0.8, in the NSC, NSC gel, and NSC‐pGNP gel groups, respectively (Figure S7e).

### 3.8 The increased synaptic connection and myelination due to the transplantation of NSC‐pGNP gel graft

The rate of Synapsin‐stained area was highest in the NSC‐pGNP gel group (20.5% ± 1.5) compared to those in the NSC (7.4% ± 0.5) and NSC gel group (8.6% ± 1.0) (Figure S8). The Synapsin is a major post‐synaptic marker.^1^ We also found that the rate of Tuj1/MBP (a myelination marker)‐stained area was highest in the NSC‐pGNP gel group (29.0% ± 2.7, Figure S9).

### 3.9 The neuronal differentiation rate of grafted NSCs was highest in the NSC‐pGNP gel group

To investigate the differentiation rate into mature neurons of the transplanted NSCs, we used GFP/Tuj1/NeuN‐stained sections (Figure S10). The rate of Tuj1/NeuN‐stained area was significantly increased in the NSC‐pGNP gel group (23.4% ± 3.5, ***p < 0.001).

We used Tuj1/OSP/GFAP/GFP‐stained sections for the evaluation of the cellular differentiation of transplanted NSCs in SCI. As shown in the Figure S11d, the rates of GFAP/GFP‐stained area were decreased in the order of the NSC, NSC gel, and NSC‐pGNP gel groups (31.3% ± 4.5, 26.1% ± 2.6, and 17.6% ± 1.4, respectively). The rates of Tuj1/GFP‐stained area were 19.3% ± 2.5, 24.6% ± 2.3, and 38.8% ± 2.6 in the NSC, NSC gel, and NSC‐pGNP gel groups, respectively (***p < 0.001, Figure S11e).

### 3.10 Axonal changes due to the transplantation of the NSC‐pGNP gel graft

As shown in the Figure S12, the axons stained with NF was increased in the NSC‐pGNP gel group (19.5% ± 2.3, ***p < 0.001) compared to that in the NSC gel group (6.3% ± 1.0). In addition, the rate of GAP43‐stained areas (Figure S13) was increased in the NSC‐pGNP gel group (23.9% ± 2.2, ***p < 0.001) compared to that in the NSC gel group (9.4% ± 1.3). The GAP43 marker is useful for the detection of the regenerated axons.^33^

### 3.11 The inhibition of microglia/macrophages due to the NSC‐pGNP gel graft

The iba1 as well as CD68 is major marker for the detection of microglia/macrophages.^34^ The rates of CD68‐stained areas around the grafted region were 15.6% ± 0.7, 5.4% ± 0.6, and 5.5% ± 0.6 in the NSC, NSC gel, and NSC‐pGNP gel groups, respectively (Figure S14d). The rates of iba1‐stained areas around the grafted region were 14.7% ± 0.9, 5.6% ± 0.5, and 5.3% ± 0.7 in the NSC, NSC gel, and NSC‐pGNP gel groups, respectively (Figure S14e).

## 4 DISCUSSION

Several studies have reported that NSC‐derived neurons promote the recovery of injured spinal cords.^35^, ^36^ However, the NSCs are limited in that they are more likely to differentiate into astrocytes than neurons under SCI conditions.^37^, ^38^, ^39^ With regard to allogeneic NSCs, their differentiation into astrocytes is greatly increased.^40^ Therefore, the establishment of an environment favoring neuronal differentiation after transplantation of NSCs is a primary issue to be considered for the recovery of injured spinal cords. According to the report of Hwang et al, the Tuj1 staining rate (of all GFP stained area) from the E14 spinal cord derived NSC was approximately 30% at 63 DPI. The rats were accompanied with physical therapy, including treadmill locomotor training for recovery.^41^ Lu et al. also showed neuronal differentiation from E14 spinal‐cord‐derived NSCs.^8^ The transplanted NSC graft contained 10 types of growth factors.^8^ In that study, the differentiation rates into neurons, oligodendrocytes, and astrocytes were 27.5% ± 2.7 (for the entire GFP stained area), 26.6% ± 3.9, and 15.9% ± 1.6, respectively, from grafted cells.

**FIGURE 8:**
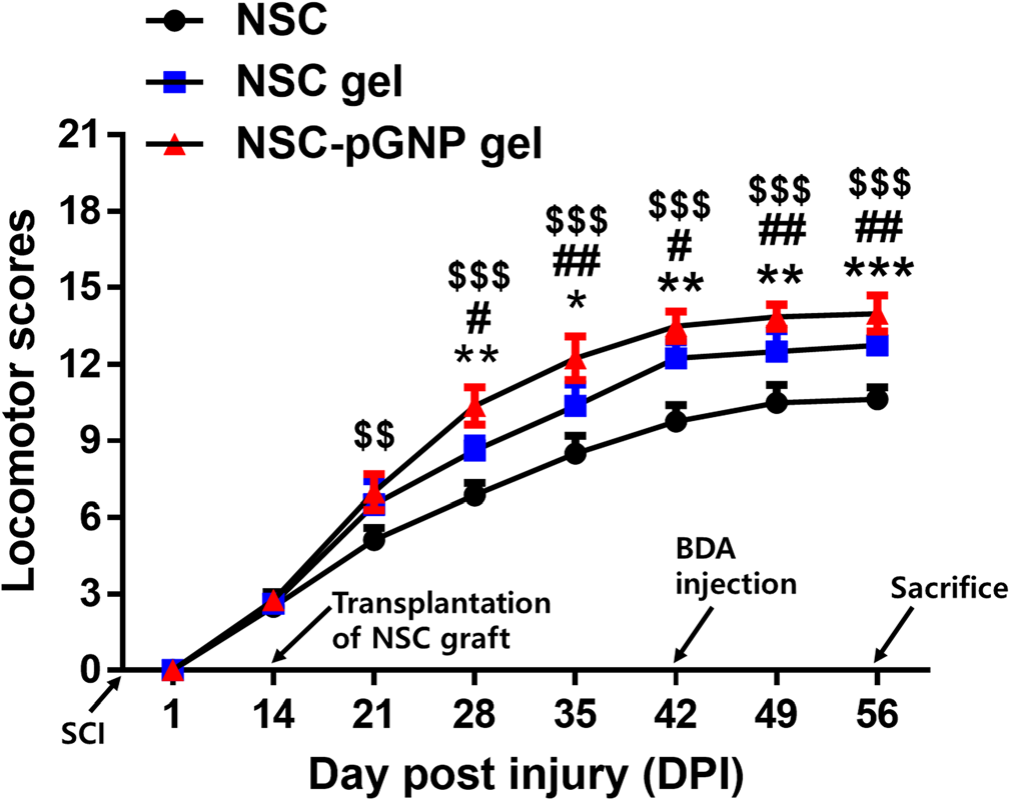
Basso–Beattie–Bresnahan (BBB) hindlimb locomotor scores of the spinal cord injury rats (*n* = 4 per group). Comparison of BBB locomotor scores in the neural stem cell (NSC), NSC gel, and NSC‐positively charged gold nanoparticles (pGNP) gel groups at 1, 14, 21, 28, 35, 42, 49, and 56‐days post injury (DPI). Results are the mean ± SD; a significant difference among the NSC, NSC gel, and the NSC‐pGNP gel groups at the same DPI was also demonstrated (NSC vs. NSC gel: *p < 0.05, **p < 0.01, and ***p < 0.001; NSC gel vs. NSC‐pGNP gel: ^#^ *p* < 0.05 and ^##^ *p* < 0.01; NSC vs. NSC‐pGNP gel: ^$$^ *p* < 0.01 and ^$$$^ *p* < 0.001). The significant differences were analyzed by a one‐way ANOVA, and Tukey's multiple‐comparison test was used as a post‐hoc analysis method

GFAP barriers in the chronic phase inhibit axon sprouting and regeneration in injured spinal cords by secreting inhibitory molecules to the lesion.^42^ This process in fact contributes to the failure of locomotor recovery following SCI. Meanwhile, multiple studies have reported that neuronal repair within injured spinal cords can improve locomotor recovery after the chronic phase.^35^, ^41^ Therefore, we focused on developing a neuron‐inducing graft that induces NSC differentiation toward neurons as opposed to astrocytes, leading to possible functional recovery in the SCI rats. Indeed, we observed significantly increased Tuj1‐stained neurons within the injury area (Figures 6b and 7b, ***p < 0.001), and the locomotor scores (Figure 8, ^##^ *p* < 0.01) in the NSC‐pGNP group are improved to those in the two control groups (NSC and NSC gel groups) at 56 DPI.

We observed that more than 50% of the pGNP was endocytosed into the NSCs at 24 h (Figure 4c) and increased cellular differentiation toward neurons (Figures 2c and 3a). This outcome demonstrated that pGNP could promote neuronal differentiation. Given that NSC‐derived neurons can secrete various neurotrophic factors,^2^ the synergetic interaction between the neurons and pGNP may further increase the differentiation of NSCs into neurons despite SCI conditions (Figures 6b and 7b). BDA is widely used to trace axons in spinal cords.^1^ In this study, the injured axons in the NSC (Figure S5d) and NSC gel (Figure S6d) groups were rarely recovered after the injury. However, we observed significant increases in the numbers of axons within the injury area in the NSC‐pGNP group (7.0 ± 0.8, Figure 7d, ***p < 0.001). These results indicate that pGNP is a key material for the recovery of an injured spinal cord. Indeed, GNPs are promising materials when used to recover the functions of an injured spinal cord.

In our study, more than 50% of the pGNP was endocytosed into the cells within 24 h (Figure 4c). Wei et al. showed the increased neuronal differentiation of embryonic stem cells according to the increased GNP‐endocytosis.^43^ The GNPs onto cell membranes was reported to endocytose into the cells through the receptor‐mediated endocytosis, including clathrin‐mediated endocytosis.^44^ However, further studies are warranted whether endocytosis must be needed for neuronal differentiation.

Our results demonstrated that both nGNP and pGNP increased the differentiation rate into neurons from embryo derived NSCs in vitro. Specifically, the neuronal differentiation rate was higher in the pGNP group than that in the nGNP group. In vivo, the NSCs in the NSC‐pGNP gel group were noticeably differentiated into neurons than those in the two types of control groups (NSC and NSC gel groups). The highest recovery rate of injured axons in the transplanted region was also shown in the NSC‐pGNP gel group. These results indicate that the neuron‐inducing pGNP gel graft are promising as a mediator for the recovery of injured spinal cords.

## 5 CONCLUSION

The primary goal in the present study was the establishment of a means by which to maintain a neuron‐inducing environment after the transplantation of NSC grafts under SCI conditions. The rate of cellular differentiation into neurons from E14 spinal‐cord‐derived NSCs was markedly increased in the NSC‐pGNP gel group compared to those in both the NSC and NSC gel groups despite SCI conditions. Based on our outcomes, we suggest that the neuron‐inducing NSC‐pGNP gel can be applied as a stem cell therapy after SCI.

## CONFLICT OF INTEREST

The authors declare no competing financial interest.

## AUTHOR CONTRIBUTIONS

**Wan‐Kyu Ko:** Conceptualization (lead); validation (equal); writing – original draft (lead). **Seong Jun Kim:** Investigation (lead); methodology (equal); validation (equal). **Gong Ho Han:** Investigation (equal); methodology (lead). **Daye Lee:** Validation (equal); visualization (lead). **Dabin Jeong:** Data curation (lead). **Sang Jin Lee:** Formal analysis (lead). **In‐Bo Han:** Validation (lead). **Je Beom Hong:** Resources (lead). **Seung Hun Sheen:** Software (lead). **Seil Sohn:** Funding acquisition (lead); project administration (lead); supervision (lead); writing – review and editing (lead).

## Supporting information


**Appendix S1**Supporting information

## Data Availability

The data that support the findings of this study are available from the corresponding author upon reasonable request.
