# Supplementary material for: Transplantation of neuron‐inducing grafts embedding positively charged gold nanoparticles for the treatment of spinal cord injury
Source: Bioeng Transl Med. 2022 Apr 18;7(3):e10326. doi: 10.1002/btm2.10326 (PMC9472004; doi:10.1002/btm2.10326)
Supplement: Supplementary file 1 — Appendix S1Supporting information [file BTM2-7-e10326-s001.pdf]

Supporting Information

Transplantation of neuron-inducing grafts embedding positively charged gold nanoparticles for the treatment of spinal cord injury

Wan-Kyu Ko[1,2], Seong Jun Kim[1,2], Gong Ho Han[1,2], Daye Lee[1,2], Dabin Jeong[1,3], Sang Jin Lee[4], In-Bo Han[1], Je Beom Hong[5], Seung Hun Sheen[1], and Seil Sohn[1,*]

[1]Department of Neurosurgery, CHA Bundang Medical Center, CHA University, 59 Yatap-ro, Bundang-gu, Seongnam-si, Gyeonggi-do, 13496, Republic of Korea

[2]Department of Biomedical Science, CHA Bundang Medical Center, CHA University, 335 Pangyo-ro, Bundang-gu, Seongnam-si, Gyeonggi-do, 13488, Republic of Korea

[3]Department of Biology, Lawrence University, 711 E. Boldt Way, Appleton, WI 54911, USA

[4]Department of Dental Materials, School of Dentistry, Kyung Hee University, 26 Kyungheedae-ro, Dongdaemun-gu, Seoul, 02447, Republic of Korea

[5]Department of Neurosurgery, Kangbuk Samsung Hospital, Sungkyunkwan University School of Medicine, 29 Saemunan-ro, Jongno-gu, Seoul, 03181, Republic of Korea

1. MATERIALS AND METHODS

1.1. Preparation of nGNP

$HAuCl_4$ powder (136 mg) was dissolved in 800 ml of DW. Three hundred mg of citrate powder were mixed with 15 ml of DW to make citrate solution. The $HAuCl_4$ solution was refluxed at 110 °C and the citrate solution (15 ml) was quickly added to the $HAuCl_4$ solution. After 20 min, the color of the solution became dark red, containing approximately 30 nm of negative GNPs. The concentration of synthesized nGNP was 0.52 nM, which was estimated by the Beer-Lambert law with a molar extinction coefficient of 3.36×10^9 $M^{-1}cm^{-1}$.

1.2. Preparation of 2% gC and 3% oHA

The oHA was synthesized through an oxidation process using sodium periodate ($NaIO_4$). The HA (3.8 g) was dissolved in DW (360 ml). The sodium periodate (1.068 g) was dissolved in another amount of DW (40 ml). The dissolved sodium periodate was slowly added to the HA in a dark room. The mixture was stirred for 24 h and 1 mL of ethylene glycol (Sigma) was added to the mixture to neutralize the unreacted sodium periodate. The resultant solution was dialyzed for seven days using dialysis membranes (Spectrum Spectra, molecular weight cut off (MWCO): 12–14 K). After dialysis, the oHA solution was lyophilized. The 2% gC and 3% oHA were separately dissolved in DPBS and centrifuged to remove the bubbles for 30 seconds at 13,000 RPM. These samples were stored at – 20 °C before the use.

1.3. qRT-PCR

NSCs (4×10^6) embedded with 20 µl of hydrogels (n = 3 per group) were cultured with none (for gel group), 0.1 nM nGNP (for nGNP gel group), or 0.1 nM pGNP (for pGNP gel group). The NSC gels were cultured with medium for six days. The RNA in the groups was extracted using Trizol reagent

(Invitrogen) according to the manufacturer's instructions. Complementary DNA (cDNA) was synthesized from 1 μg of total RNA using a Maxim RT Premix kit (iNtRON Biotechnology, Inc., Korea). The qRT-PCR step was performed with an ABI Step-One real-time PCR system (Applied Biosystems, Warrington, UK). The reaction mixture consisted of the SYBR Green 2X PCR Master Mix (Applied Biosystems), a cDNA template, and forward/reverse primers. The relative expression levels of Tuj1, GFAP, and Olig2 were normalized to that of GAPDH using the $2^{-\Delta\Delta CT}$ method. The primers were obtained from Bioneer (Daejeon, Korea). The primer sequences used in this study are shown in Table S1. The experiments were conducted in triplicate.

1.4. Tissue preparation and IF staining for the measurement of the injury area

Two sections (5 μm thick) per specimen (n = 8 per group) were stained with anti-CD68/GFAP. The injury area surrounded an astrocytic barrier (stained with GFAP, mouse, Abcam at 1:200) is indicated by the white dashed lines. The ROI areas (composite tiled scan images, 4,500 × 2,000 μm^2, scale bar: 500 μm) were normalized to 100% and relative injury areas were quantified. For the quantification of CD68 (rabbit, Abcam at 1:200 at 1:200) stained area, the injury area was normalized to 100% and the CD68-stained areas within the injury areas were quantified using ImageJ software (NIH). Alexa 488/568-secondary antibodies (1:500, Invitrogen) were used and the sections were detected through a confocal laser-scanning microscope (LSM 880, Carl Zeiss).

1.5. GFP/Tuj1/Syn and GFP/Tuj1/MBP staining

Two sections (5 μm thick) per specimen (n = 8 per group) were stained with anti-GFP/Tuj1/Syn and anti-GFP/Tuj1/MBP. The injury area within the designated ROI (composite tiled scan images, 4,500 × 2,000 μm^2, scale bar: 500 μm) was normalized to 100%. The Tuj1/Syn and Tuj1/MBP-stained areas were quantified using ImageJ software (NIH). The GFP (chicken, Abcam at 1:200), Tuj1 (mouse, Abcam at 1:200), Syn (rabbit, Abcam at 1:200), and MBP (rabbit, Abcam at 1:200) antibodies were

conjugated with Alexa 488 (for GFP), 568 (for Tuj1), and 647 (for Syn or MBP)- secondary antibodies (1:500, Invitrogen).

1.6. GFP/Tuj1/NeuN and Tuj1/OSP/GFAP/GFP staining

Two sections (5 μm thick) per specimen (n = 8 per group) were stained with anti-GFP/Tuj1/NeuN and anti-Tuj1/OSP/GFAP/GFP. The injury area within the designated ROI (composite tiled scan images, 4,500 × 2,000 μm^2, scale bar: 500 μm) was normalized to 100%. The Tuj1/NeuN, GFAP/GFP, Tuj1/GFP, and OSP/GFP-stained areas were quantified using ImageJ software (NIH). The GFP (Guinea pig, CosmoBio at 1:200), Tuj1 (mouse, Abcam at 1:200), NeuN (rabbit, Abcam at 1:200), and GFAP (chicken, Abcam at 1:200) antibodies were conjugated with Alexa 488 (for GFP), 568 (for Tuj1), 594 (for OSP) and 647 (for NeuN or GFAP)-secondary antibodies (1:500, Invitrogen).

1.7. GFP/NF/PGP9.5 and GFP/Tuj1/GAP43 staining

Two sections (5 μm thick) per specimen (n = 8 per group) were stained with anti-GFP/NF/PGP9.5 and anti-GFP/Tuj1/GAP43. The injury area within the designated ROI (composite tiled scan images, 4,500 × 2,000 μm^2, scale bar: 500 μm) was normalized to 100%. The NF, PGP9.5, and GAP43-stained areas were quantified using ImageJ software (NIH). The GFP (chicken, Abcam at 1:200), Tuj1 (mouse, Abcam at 1:200), NF (rabbit, Abcam at 1:1,000), PGP9.5 (guinea pig, Abcam at 1:200), and GAP43 (rabbit, Abclonal at 1:200) antibodies were conjugated with Alexa 488 (for GFP), 568 (for PGP9.5 or Tuj1), and 647 (for NF or GAP43)-secondary antibodies.

1.8. GFP/CD68/iba1 staining

Two sections (5 μm thick) per specimen (n = 8 per group) were stained with anti-GFP/CD68/iba1. The injury area within the designated ROI (composite tiled scan images, 4,500 × 2,000 μm^2, scale bar: 500 μm) was normalized to 100%. The CD68 and iba1-stained areas were quantified using ImageJ software

(NIH). The GFP (chicken, Abcam at 1:200), CD68 (mouse, Abcam at 1:200), iba1 (rabbit, Abcam at 1:200) antibodies were conjugated with Alexa 488 (for GFP), 568 (for CD68), and 647 (for iba1)-secondary antibodies.

Supporting Figures

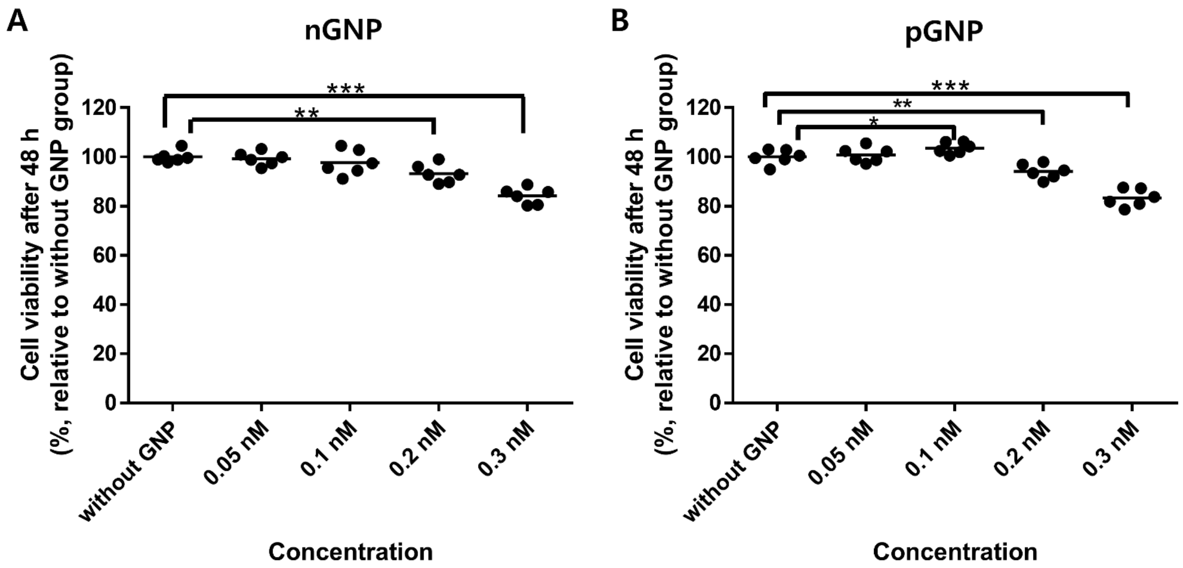

Figure S1. The NSCs were treated with various concentrations of nGNP or pGNP (0, 0.05, 0.1, 0.2, or 0.3 nM in both cases) for 48 h. NSC viability of the (A) nGNP or (B) pGNP-treated groups was measured using a CCK-8 assay. Results are the mean ± SD: *p < 0.05, **p < 0.01 and ***p < 0.001; significant differences between outcomes without GNPs and each concentration of GNPs were analyzed by Student's t-tests (n = 6).

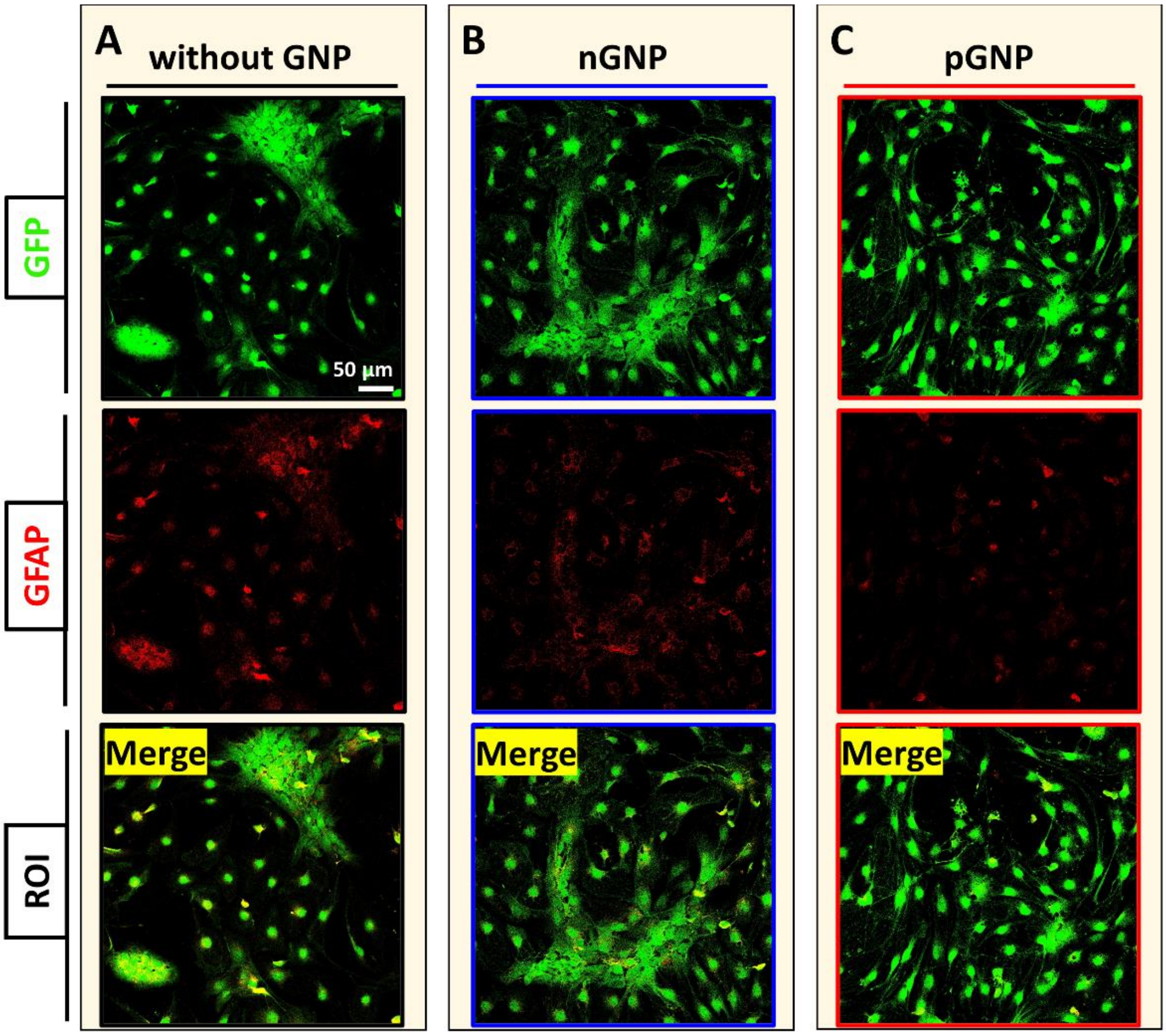

Figure S2. Cellular differentiation of GFP-expressing NSCs. The cells were cultured for six days without GNP, with nGNP (0.1 nM), or with pGNP (0.1 nM). On day 6, the cells were co-stained with GFP and GFAP. Representative images (also designated as the ROIs, 425 × 425 μm^2) of the groups (A) without GNP, (B) with nGNP, and (C) with pGNP were demonstrated at 20 × magnification. Scale bar is 50 μm.

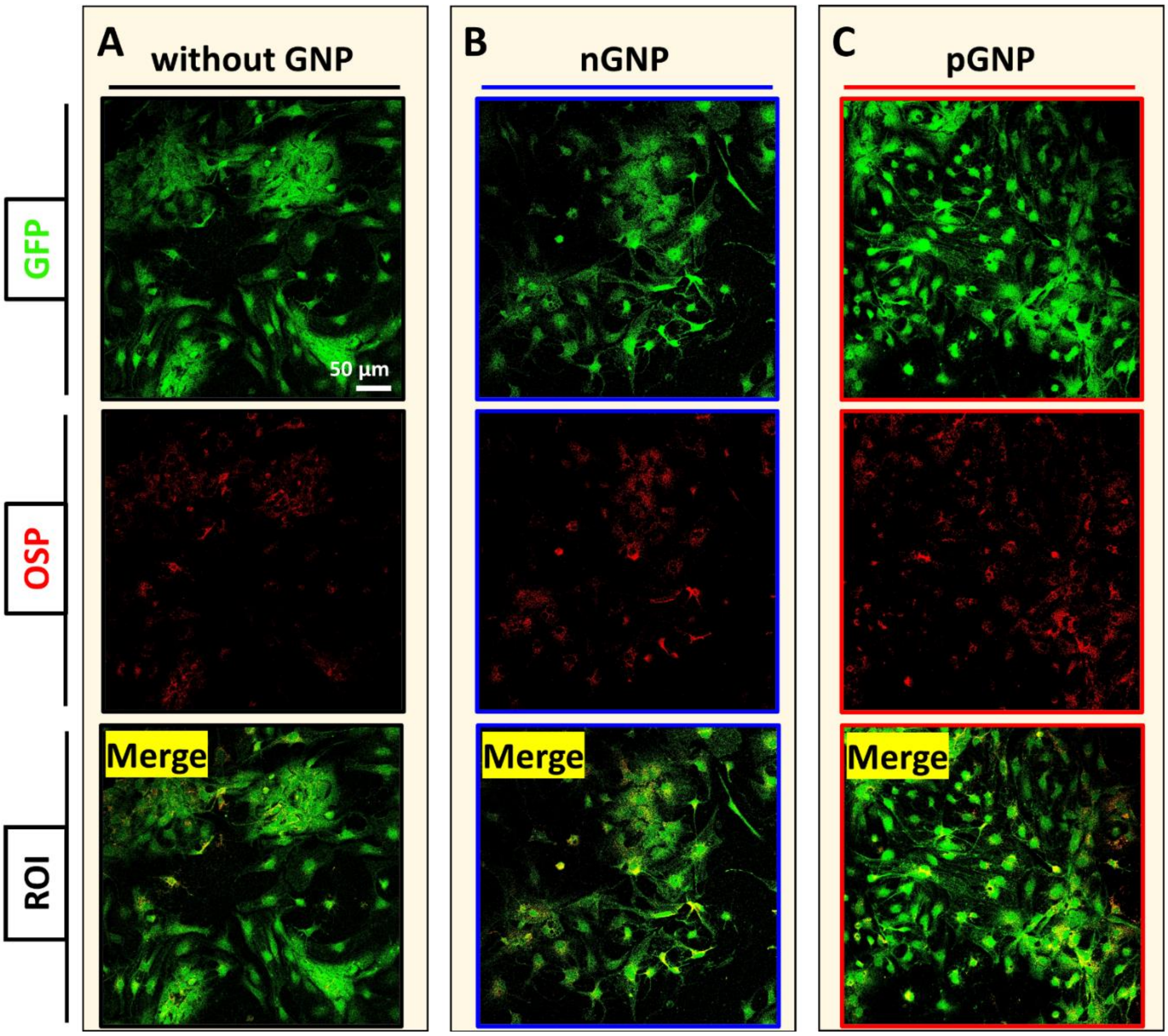

Figure S3. Cellular differentiation of GFP-expressing NSCs. The cells were cultured for six days without GNP, with nGNP (0.1 nM), or with pGNP (0.1 nM). On day 6, the cells were co-stained with GFP and OSP. Representative images (also designated as the ROIs, 425 × 425 μm^2) of the groups (A) without GNP, (B) with nGNP, and (C) with pGNP were demonstrated at 20 × magnification. Scale bar is 50 μm.

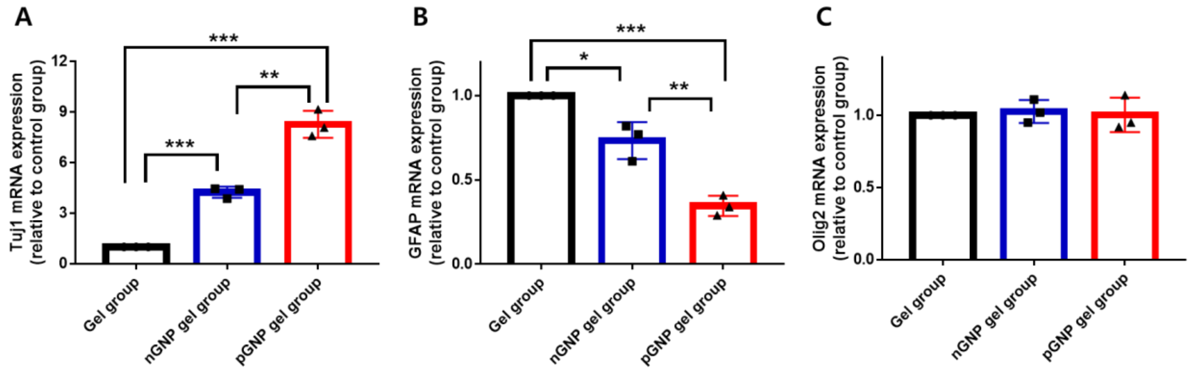

Figure S4. The (A) Tuj1, (B) GFAP, and (C) Olig2 mRNA expression values in the gel, nGNP gel, and pGNP gel groups. The results are expressed as the mean ± SD (n = 3 per group) and the experiments was conducted in triplicate. Differences with *$p < 0.05$, **$p < 0.01$, and ***$p < 0.001$ were considered statistically significant.

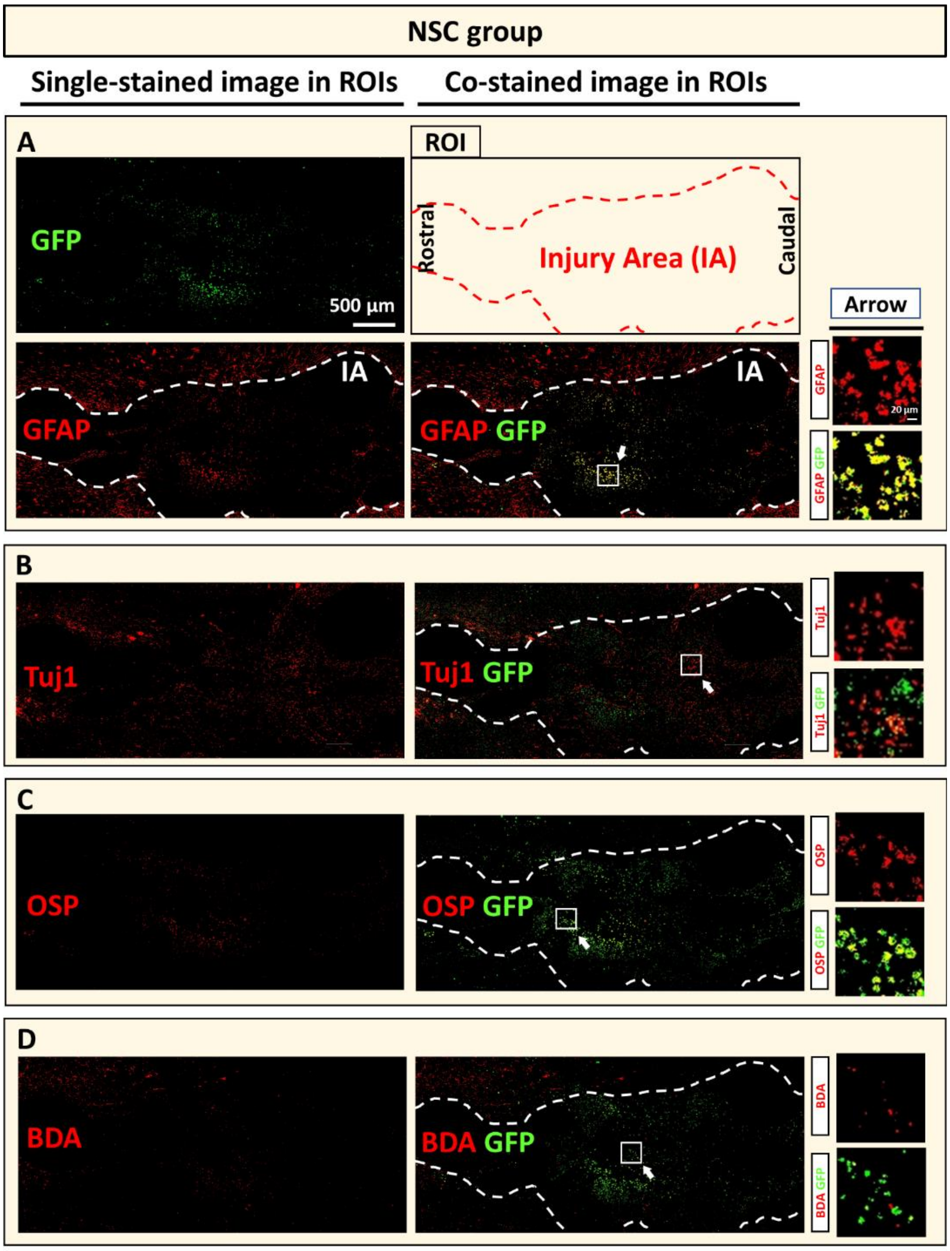

Figure S5. Cellular differentiation of grafted NSCs under the SCI condition for the NSC group. Horizontally sectioned specimens were stained with the GFP antibody. Each GFP-stained section was co-stained with GFAP (36th and 40th), Tuj1 (37th and 41st), OSP (38th and 42nd), or biotinylated dextran amines (BDA, 39th and 43rd). Representative tile scan images (also designated as ROIs, 4,500 × 2,000 μm^2) of samples co-labelled with (A) GFAP/ GFP, (B) Tuj1/GFP, (C) OSP/ GFP, and (D) BDA/GFP are shown. Scale bar of the ROI image is 500 μm. Arrows indicate randomly designated regions for higher magnification views. The designated region is 170 × 170 μm^2 (Scale bar: 20 μm).

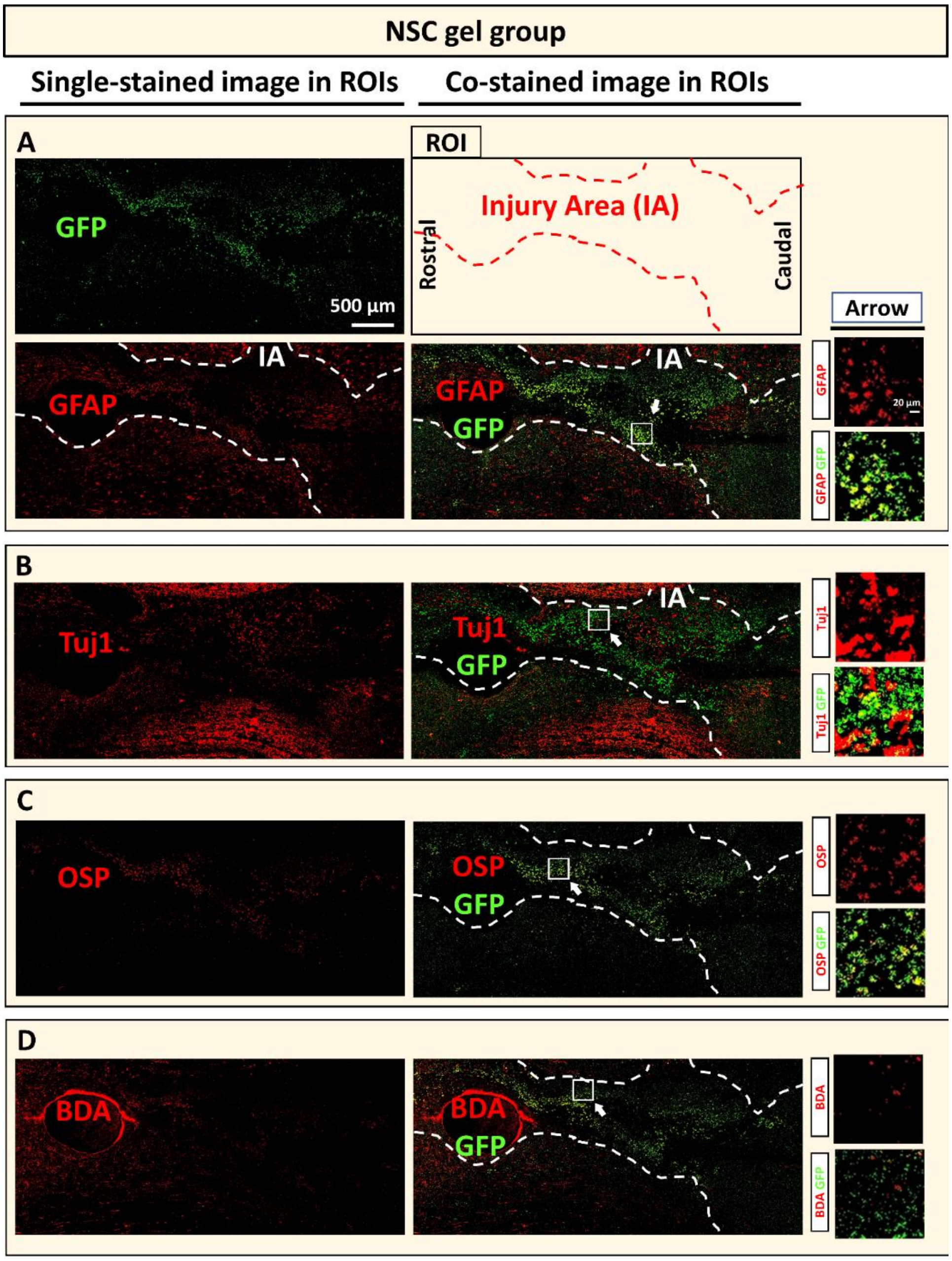

Figure S6. Cellular differentiation of grafted NSCs under the SCI condition for the NSC gel group. Horizontally sectioned specimens were stained with the GFP antibody. Each GFP-stained section was co-stained with GFAP (36th and 40th), Tuj1 (37th and 41st), OSP (38th and 42nd), or biotinylated dextran amines (BDA, 39th and 43rd). Representative tile scan images (also designated as ROIs, 4,500 × 2,000 µm^2) of samples co-labelled with (A) GFAP/ GFP, (B) Tuj1/GFP, (C) OSP/ GFP, and (D) BDA/GFP are shown. Scale bar of the ROI image is 500 µm. Arrows indicate randomly designated regions for higher magnification views. The designated region is 170 × 170 µm^2 (Scale bar: 20 µm).

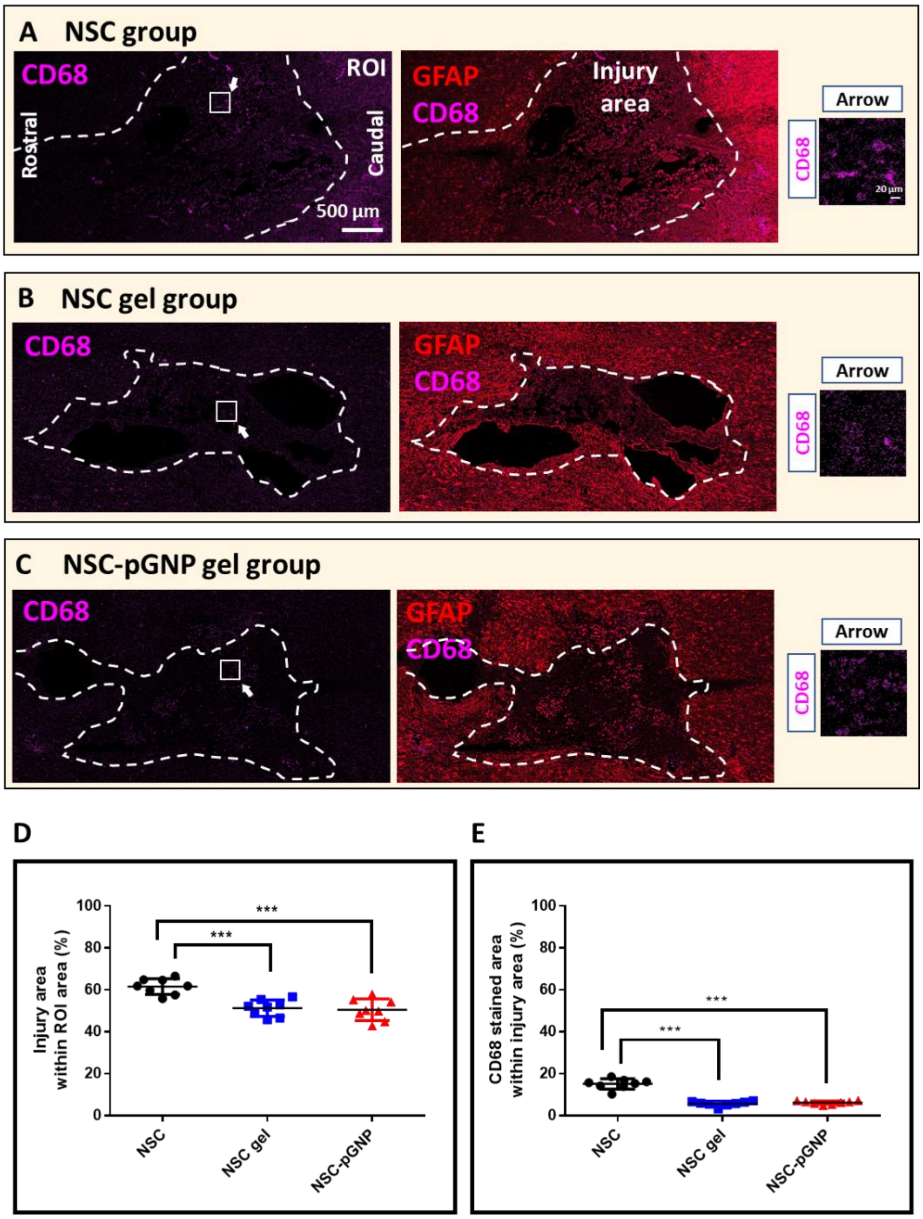

Figure S7. Representative tile scan images (4,500 × 2,000 μm^2) stained with CD68/GFAP for the (A) NSC, (B) NSC gel, (C) NSC-pGNP gel groups are shown. Scale bar of the tile scan image is 500 μm. Arrows indicate randomly designated regions for higher magnification views. The designated region is 170 × 170 μm^2 (Scale bar: 20 μm). (D) Injury areas surrounded with GFAP barriers and (E) CD68-stained areas within the injury area were quantified (n = 8 per group). Results are the mean ± SD: ***p < 0.001; significant differences among the three groups were demonstrated.

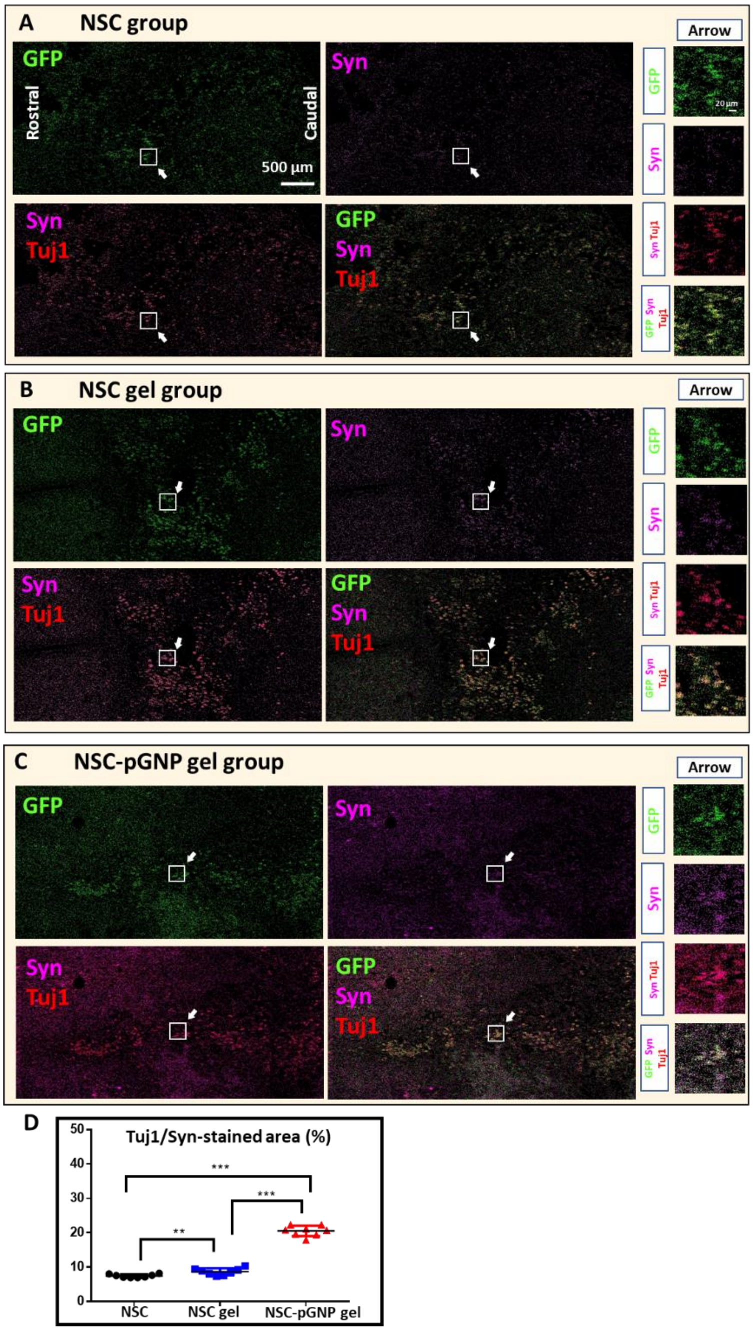

Figure S8. Representative tile scan images (4,500 × 2,000 µm^2) stained with GFP/Tuj1/Synapsin (Syn) for the (A) NSC, (B) NSC gel, (C) NSC-pGNP gel groups are shown. Scale bar of the tile scan image is 500 µm. Arrows indicate randomly designated regions for higher magnification views. The designated region is 170 × 170 µm^2 (Scale bar: 20 µm). (D) Tuj1/Syn-stained area within injury areas were quantified (n = 8 per group). Results are the mean ± SD: $^{**}p < 0.01$ and $^{***}p < 0.001$; significant differences among the three groups were demonstrated.

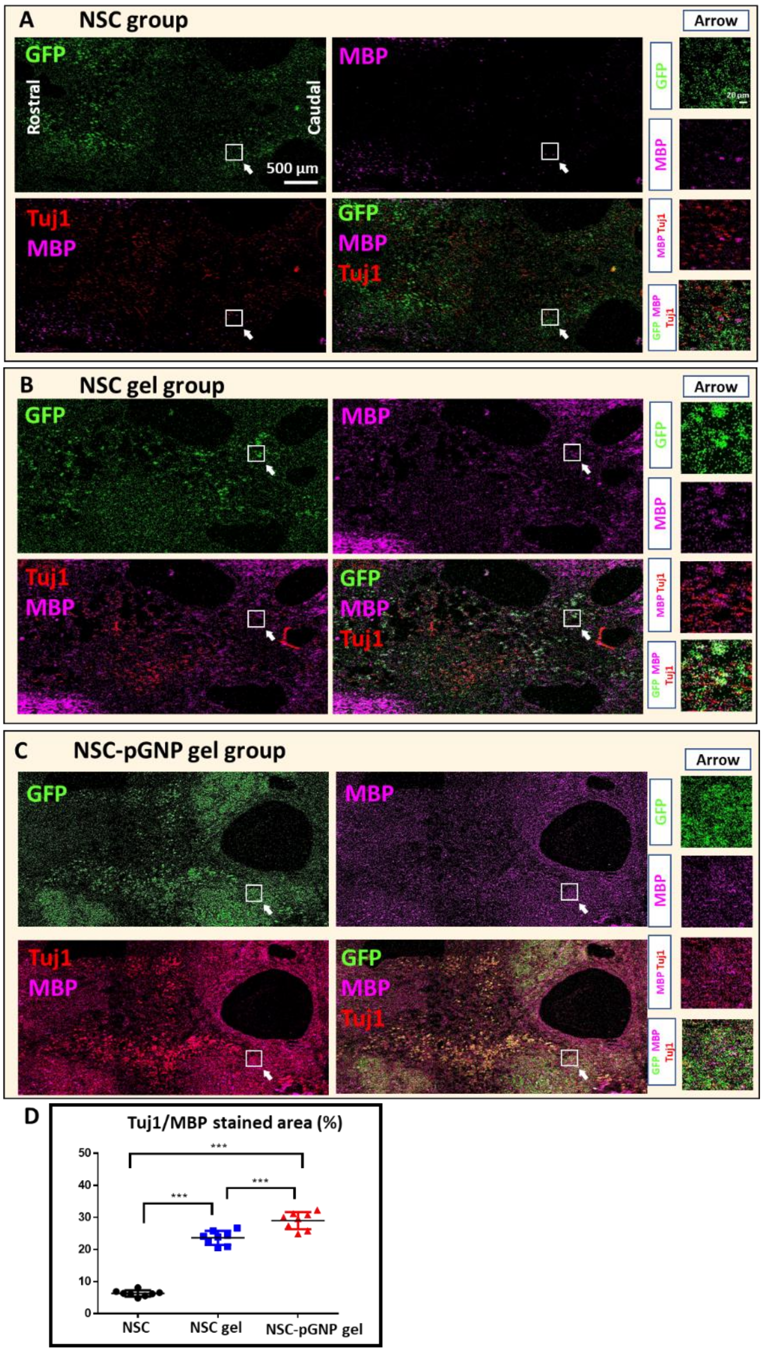

Figure S9. Representative tile scan images (4,500 × 2,000 μm^2) stained with GFP/Tuj1/MBP for the (A) NSC, (B) NSC gel, (C) NSC-pGNP gel groups are shown. Scale bar of the tile scan image is 500 μm. Arrows indicate randomly designated regions for higher magnification views. The designated region is 170 × 170 μm^2 (Scale bar: 20 μm). (D) Tuj1/MBP-stained area within injury areas were quantified (n = 8 per group). Results are the mean ± SD: ***$p < 0.001$; significant differences among the three groups were demonstrated.

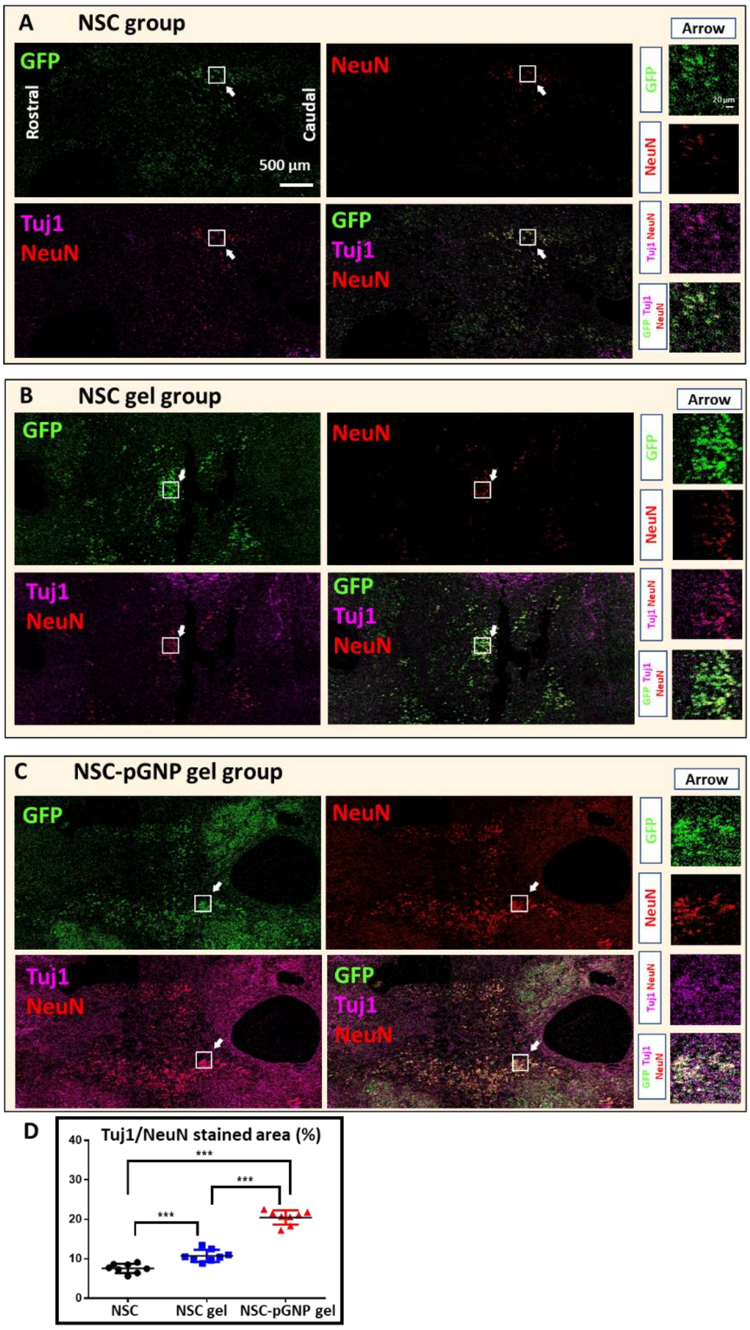

Figure S10. Representative tile scan images (4,500 × 2,000 μm^2) stained with GFP/Tuj1/NeuN for the (A) NSC, (B) NSC gel, (C) NSC-pGNP gel groups are shown. Scale bar of the tile scan image is 500 μm. Arrows indicate randomly designated regions for higher magnification views. The designated region is 170 × 170 μm^2 (Scale bar: 20 μm). (D) Tuj1/NeuN-stained area within injury areas were quantified (n = 8 per group). Results are the mean ± SD: ***$p < 0.001$; significant differences among the three groups were demonstrated.

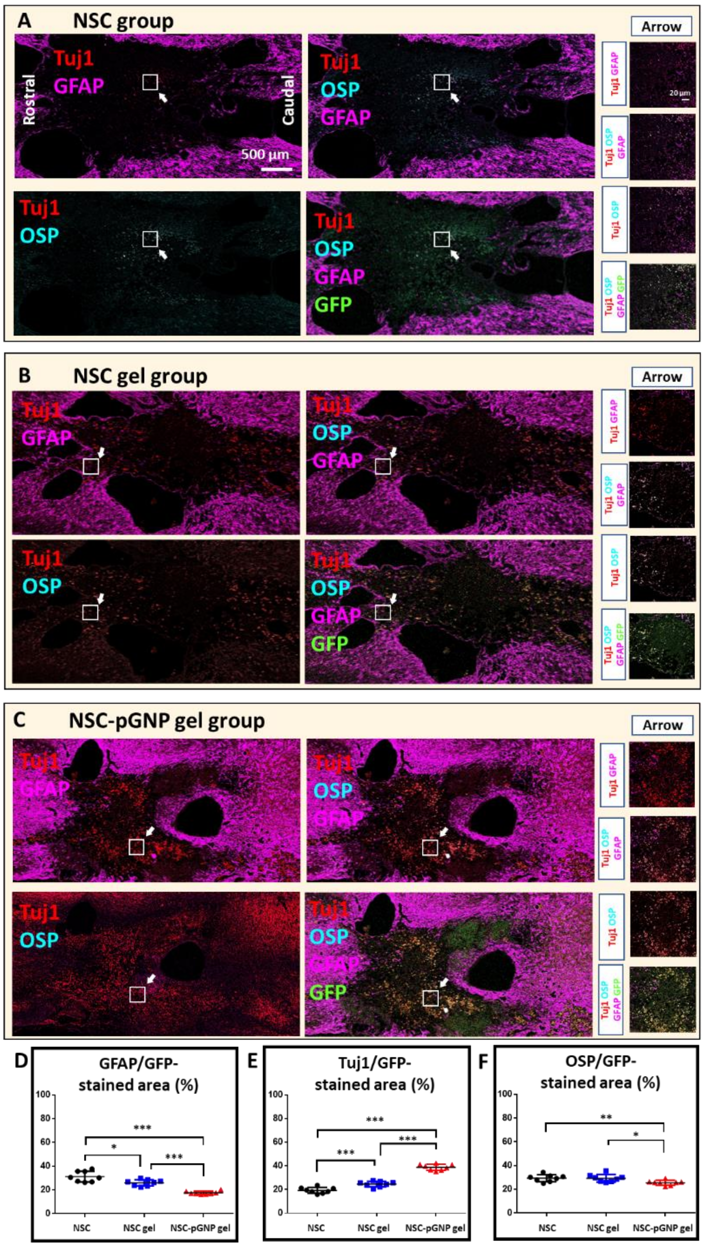

Figure S11. Representative tile scan images (4,500 × 2,000 μm^2) stained with Tuj1/GFAP, Tuj1/OSP, Tuj1/OSP/GFAP, and Tuj1/OSP/GFAP/GFP for the (A) NSC, (B) NSC gel, (C) NSC-pGNP gel groups are shown. Scale bar of the tile scan image is 500 μm. Arrows indicate randomly designated regions for higher magnification views. The designated region is 170 × 170 μm^2 (Scale bar: 20 μm). (D) GFAP/GFP, (E) Tuj1/GFP, and (F) OSP/GFP-stained areas within injury areas were quantified (n = 8 per group). Results are the mean ± SD: *$p < 0.05$, **$p < 0.01$, and ***$p < 0.001$; significant differences among the three groups were demonstrated.

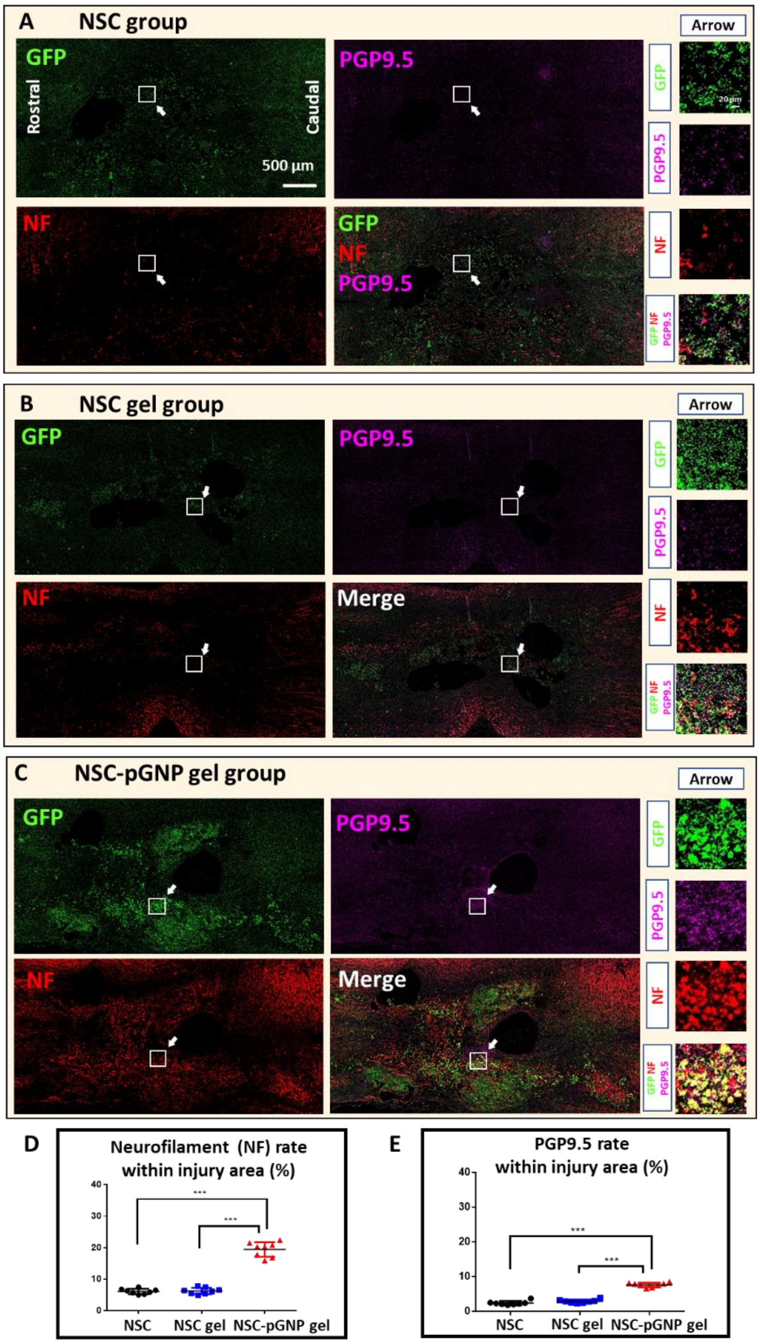

Figure S12. Representative tile scan images (4,500 × 2,000 µm^2) stained with GFP/NF/PGP9.5 for the (A) NSC, (B) NSC gel, (C) NSC-pGNP gel groups are shown. Scale bar of the tile scan image is 500 µm. Arrows indicate randomly designated regions for higher magnification views. The designated region is 170 × 170 µm^2 (Scale bar: 20 µm). (D) NF-stained area and (E) PGP9.5-stained area within injury areas were quantified (n = 8 per group). Results are the mean ± SD: ***p < 0.001; significant differences among the three groups were demonstrated.

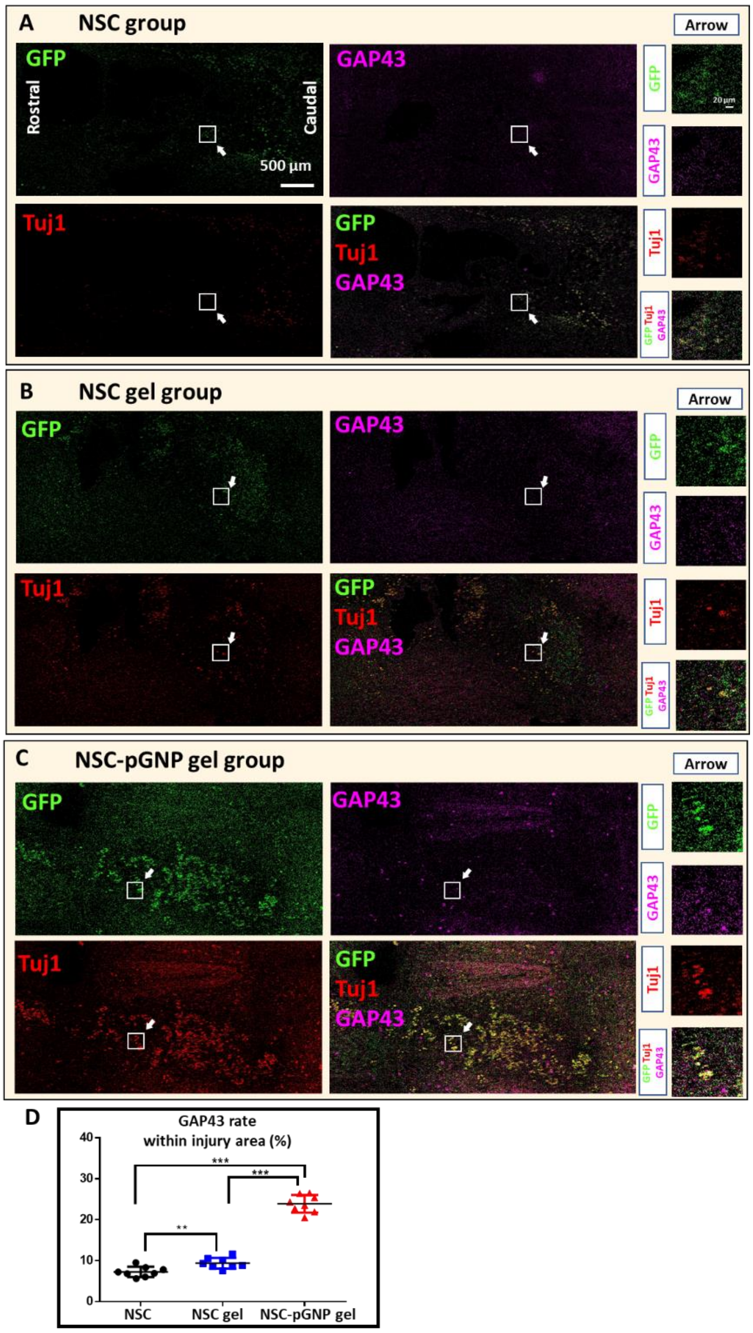

Figure S13. Representative tile scan images (4,500 × 2,000 μm^2) stained with GFP/Tuj1/GAP43 for the (A) NSC, (B) NSC gel, (C) NSC-pGNP gel groups are shown. Scale bar of the tile scan image is 500 μm. Arrows indicate randomly designated regions for higher magnification views. The designated region is 170 × 170 μm^2 (Scale bar: 20 μm). (D) GAP43-stained area within injury areas was quantified (n = 8 per group). Results are the mean ± SD: ***$p < 0.001$; significant differences among the three groups were demonstrated.

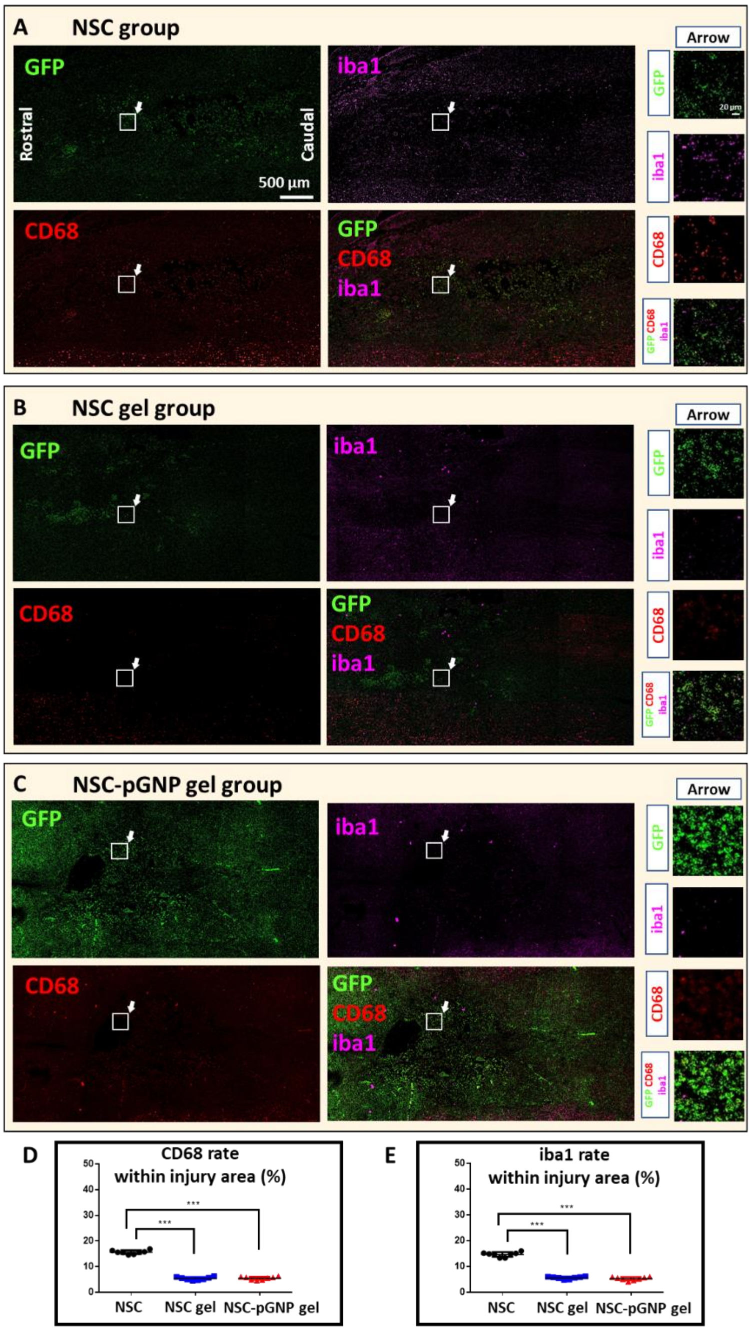

Figure S14. Representative tile scan images (4,500 × 2,000 μm^2) stained with GFP/CD68/iba1 for the (A) NSC, (B) NSC gel, (C) NSC-pGNP gel groups are shown. Scale bar of the tile scan image is 500 μm. Arrows indicate randomly designated regions for higher magnification views. The designated region is 170 × 170 μm^2 (Scale bar: 20 μm). (D) CD68 and (E) iba1-stained area within injury areas was quantified (n = 8 per group). Results are the mean ± SD: ***p < 0.001; significant differences among the three groups were demonstrated.

Table S1. Nucleotide sequences of primers used in qRT-PCR.

Gene	Forward (5' - 3')	Reverse (5' - 3')
Tuj1	GCGATGCTACCGCAAAGAAC	ATGGCGTCTCCCACACTACC
GFAP	CTGGTGTGGAGTGCCTTCGT	CACCAACCAGCTTCCGAGAG
Olig2	TTGATGAGACCGGGTTCCCT	GTCCGTGGACGTTTGCTTCTT
GAPDH	GCCATCAACGACCCCTTCAT	TTCACACCCATCACAAACA
